# Programming rumen microbiome development in calves with the anti-methanogenic compound 3-NOP

**DOI:** 10.1186/s42523-024-00343-2

**Published:** 2024-10-25

**Authors:** Gonzalo Martinez-Fernandez, Stuart E. Denman, Nicola Walker, Maik Kindermann, Christopher S. McSweeney

**Affiliations:** 1 CSIRO, Agriculture and Food, Queensland Bioscience Precinct, St. Lucia, Qld, Australia; 2 Animal Nutrition and Health, DSM Nutritional Products, Basel, Switzerland

**Keywords:** Early life intervention, Methane, Ruminants, Rumen microbial communities

## Abstract

The aim of this study was to establish a distinctive rumen microbial and fermentation profile using the anti-methanogenic compound 3-NOP to assess dam effect, and nutritional intervention of the juvenile offspring on microbial structure and function of rumen up to 12 months of age, once the treatment was withdrawn. Forty-eight pregnant heifers (H) and their future offspring (C) were allocated to either Control (-) or 3-NOP (+) treatment resulting in four experimental groups: H+/C+, H+/C-, H-/C + and H-/C-. Animals were treated from 6 weeks prior to calving until weaning, with the offspring monitored until 12 months of age. Rumen fluid samples and methane measurements using the Greenfeed system were collected during the trial. Results supported the mode of action of the compound, with a shift in fermentation from acetate to propionate, increases in branched chain fatty acids and formic acid in the 3-NOP treated animals. Similar shifts in microbial populations occurred in 3-NOP treated animals with lower abundances of rumen methanogen populations, increases of bacterial groups *Succiniclasticum spp*, *Candidatus Saccharimonas*. *Fibrobacter* and the families *Prevotellaceae and Succinivibrioacea*. and the protozoa *Entodinium*. Early life intervention had an enduring impact on the rumen microbial structure of young animals up to 28 weeks post weaning, however the effect was diminished once 3-NOP was withdrawn. Interestingly, a group of young animals emitted significantly less methane (15%) than the animals that did not receive the treatment during their juvenile stage. Our results suggest a higher resemblance of the young calf microbiome to a low methane adult and that early life colonisation of the rumen persists through to later life with the pre-weaning microbiome comprising ~ 65% of the yearling animal. Further research needs to be performed to determine the timing and dose of 3-NOP for new-born calves that can sustain a reduction in methane emissions after the treatment is withdrawn, under extensive grazing or controlled conditions.

**Supplementary Information:**

The online version contains supplementary material available at 10.1186/s42523-024-00343-2.

## Background

The microbial community in the rumen is characterized by a highly diverse population of bacterial, protozoal, bacteriophage, fungal and archaeal (methanogen) species. Acquisition of the intestinal and rumen microbiota begins at birth, and a stable microbial community develops by successive colonisation of the gut by key microorganisms. Several culture-based studies have shown that in young ruminants and during rumen development, ingested microbes colonise and establish in a defined and progressive sequence [1]. Methanogenic archaea and cellulolytic bacteria are found in the undeveloped rumen well before the ingestion of solid feed begins (2–4 days) and reach levels like those in adult animals around 10 days after birth [2, 3]. It was first reported that hay or grain rations fed early in life have an impact on the bacterial population that established in the rumen [4]. A recent review by Morgavi et al., [5] of molecular based studies confirmed with greater resolution that many of the functional populations of bacteria and methanogens comprising taxa resident in the adult animal appear in the first week of life and further colonisation events continue during suckling and before weaning.

Based on detailed molecular biology studies in various mammalian systems (humans as well as animals), it is now recognized that the developing microbiome exhibits a degree of plasticity during colonization of the gut, that occurs soon after birth and during lactation [6, 7]. This period provides a window of opportunity for targeted manipulation of the gut microbiome, such as nutritional interventions and/or inoculants, which ultimately could achieve a potential long-lasting impact on rumen metabolism and methane production in adult life [5, 6, 8, 9]. For example, it has been demonstrated that feeding a halogenated aliphatic hydrocarbon compound to goat does and their kids prior to weaning changed the rumen microbial structure and continued to decrease methane for up to four months in the young animals despite the additive being removed at weaning [10–12]. Likewise in cattle, calves treated from birth with the anti-methanogenic compound 3-nitroxypropanol (3-NOP) showed a different rumen microbial structure and emitted less methane at least a year after treatment ceased [13]. Thus, there is emerging data that suggests application of additives during the first few weeks of an animal’s life influences animal methane production and rumen microbial structure much later in life.

In addition, the maternal microbiome plays an important role in the microbial colonization of the newborn rumen [14, 15]. It has been hypothesized that the mother might transfer microorganisms to the offspring through its natural behaviour of licking the newborn after birth and other interactions such as suckling [5]. To further explore the dam effect on the establishment of microbiota in their offspring and colonisation in the developing rumen we used the anti-methanogenic compound 3-NOP to establish a characteristic microbiota in treated heifers and/or offspring for comparison with an un-treated rumen. The compound 3-NOP acts by specifically inhibiting the enzyme methyl coenzyme M reductase in the final step of methane formation, resulting in a decline in the methanogen population and an increase in the concentration of metabolic hydrogen in the rumen that shifts the microbial and metabolic profile to a low methane rumen phenotype [16–18].

Specifically, the project tested the hypothesis that the anti-methanogenic compound 3-NOP provided as a feed additive under grazing conditions to the dam in late pregnancy and lactation or to the suckling calf will alter the composition of the bacterial, protozoal and archaeal community and rumen metabolism of the juvenile animal to a profile which is reflective of lower methane production that persists into later life (up to 12 months of age).

## Results

### 3-NOP effect on rumen metabolites in the dam

No differences in body weight (537 vs. 538 kg, *P* = 0.954) and rumen fermentation parameters were detected between the groups of heifers at pre-treatment period (Supplementary Table 1).

**Fig. 1:**
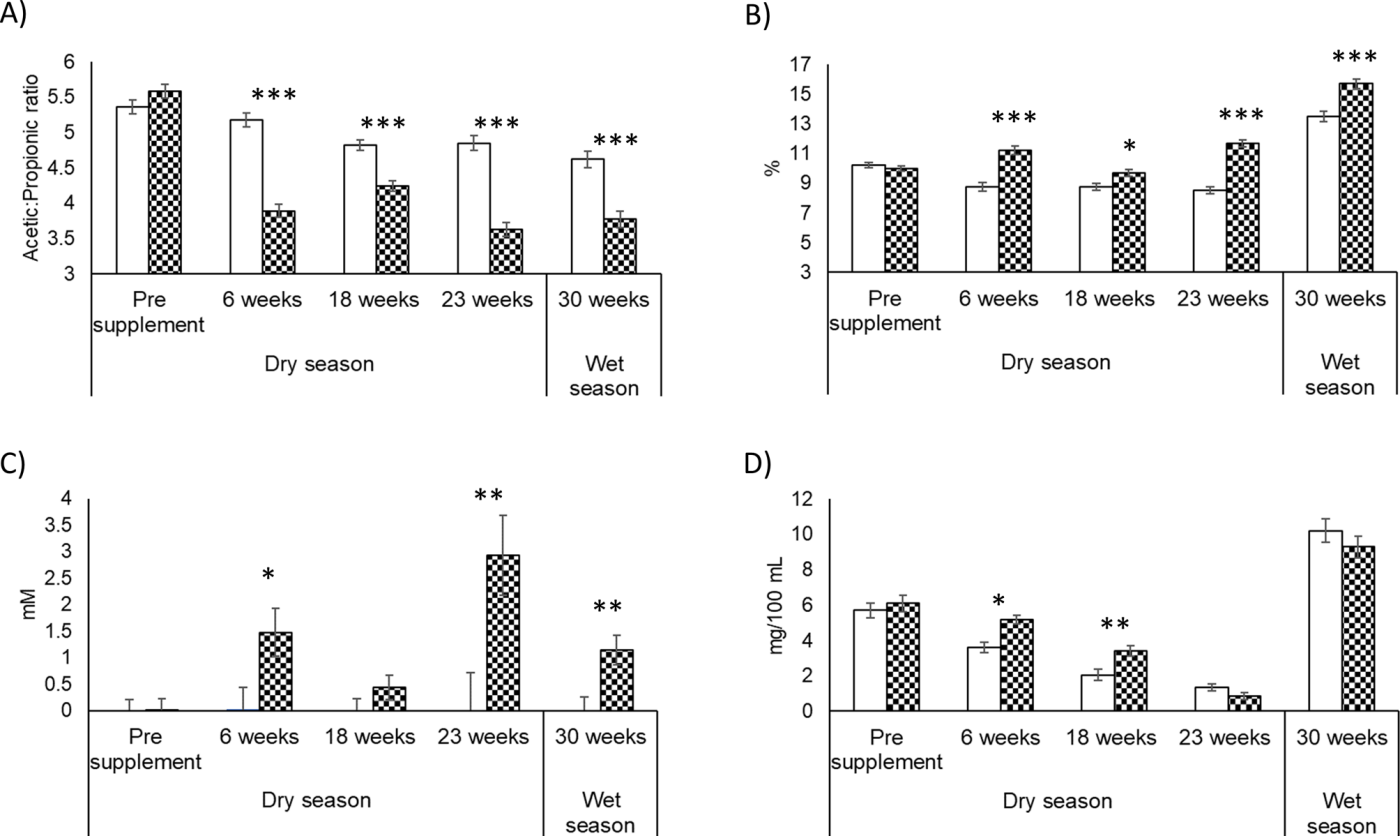
Ruminal fermentation metabolites: (**A**) acetate: propionate ratio, (**B**) butyrate, (**C**) formate and (**D**) ammonia-N in H+ (▓) or H- (□) for 30 weeks. Significant differences between 3-NOP and control (*P* < 0.001) ***, (*P* < 0.01) **, (*P* < 0.05) * for each time point

The 3-NOP effects on rumen fermentation parameters and body weight in dams from late pregnancy to the end of lactation are shown in Fig. 1 and Supplementary Tables 2– 5. No differences were observed in body weight between H + and H- groups during the trial (*P* = 0.511–0.845). Rumen VFAs showed increases (*P* < 0.05) in relative concentrations of propionate, butyrate, valerate and branched chain fatty acids and a decrease in acetate and A: P ratio in H+ (*P* < 0.001) during the whole treatment period (Fig. 1A). Ammonia concentration increased with H + compared with H- at 6 and 18 weeks (*P* = 0.015; 0.003) on treatment, which corresponded to the mid-dry season (Fig. 1D). Rumen organic acids were also affected by 3-NOP treatment, with increases in rumen formate (*P* = 0.023 − 0.005) during the whole treatment period (Fig. 1C) (except at 18 weeks on treatment (*P* = 0.181)), succinate at 23 and 30 weeks (*P* = 0.007; 0.040), and fumarate (*P* = 0.016) at 30 weeks on treatment.

### 3-NOP effect on the rumen microbiota in the dams

**Fig. 2:**
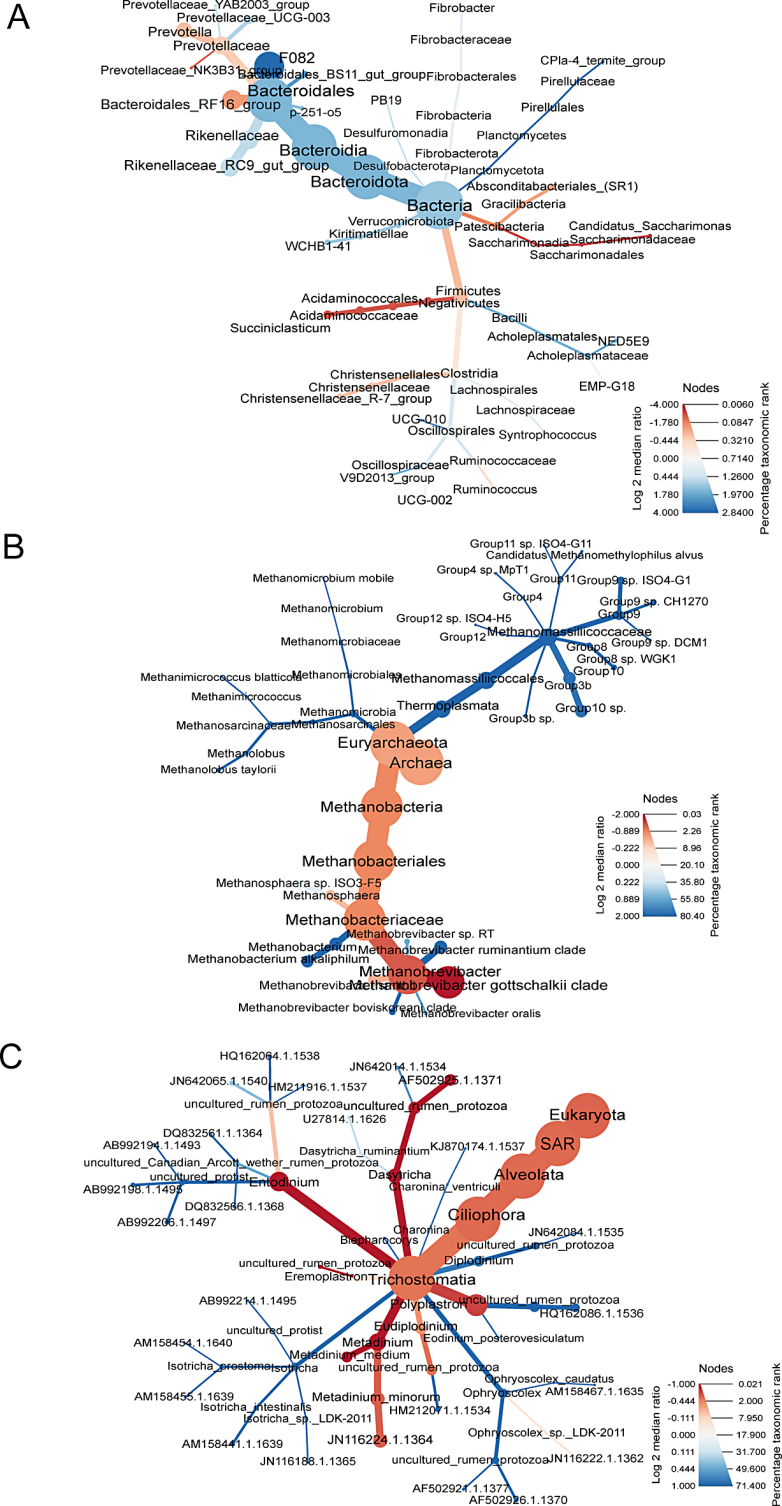
Differential taxonomic heat trees of rumen bacteria (**A**), archaea (**B**) and protozoa (**C**) communities selected by sPLS-DA showing differences in log2 median ratio between H+ (red) versus H- (blue) during the treatment period for a defined taxonomy. Node colour represents the log2 median ratio between the mean relative abundance of the taxa for each treatment, while the node size reflects the mean overall percentage of data assigned to the taxonomic rank

Using sparse PLS Discriminant Analysis (sPLS-DA), ASV’s that best characterised the dam treatment groups were determined [19]. Clustered image heatmaps (mixOmics analysis) for the selected rumen bacterial, archaeal and protozoa ASV’s at 6, 18, 23 and 30 weeks showed a distinct microbial signature for the H + compared with H- (Supplementary files 1, 2 and 3 respectively). The selected ASVs based on the sPLS-DA analysis are visualised using a differential taxonomic heat tree (Fig. 2). The bacterial ASVs (Fig. 2A) positively associated with 3-NOP classified to the family *Prevotellaceae*, *Christensenellaceae* and *Bacteroidales RF16 group* and the genera *Succiniclasticum* and *Candidatus Saccharimonas*, while phylum *Verrucomicrobiota* and families *Bacteroidales F082* and *BS11* were mainly associated with H-. The archaeal (Fig. 2B) ASVs associated with 3-NOP were classified to the family *Methanobacteriaceae*, and particularly to the species *Methanobrevibacter gottschalkii*. On the other hand, *Methanobrevibacter ruminantium*,* Methanobacterium and Methanomassiliicoccaceae family* were associated to the control animals. Regarding protozoa (Fig. 2C), ASVs classified to *Entodinium*, *Metadinium* and *Dasytricha* were positively associated with 3-NOP.

Quantitative PCR analysis of the effect of 3-NOP on the abundance of total methanogens (McrA), *Methanobrevibacter spp.* and *Methanomassiliicoccaceae* family are shown in Supplementary Fig. 2. The methanogen abundance decreased (3–6 folds) during the treatment period for H + compared with H- (*P* < 0.002), except at 23 weeks of treatment (*P* = 0.083). The *Methanomassiliicoccaceae* family was decreased (3–5 folds) only for H + at 23 and 30 weeks (*P* = 0.008; 0.001), while *Methanobrevibacter* was decreased (5-fold) for H + at 6 weeks (*P* = 0.001) compared with the H-.

### Rumen metabolites and methane production in offspring

#### 3-NOP effect

**Fig. 3:**
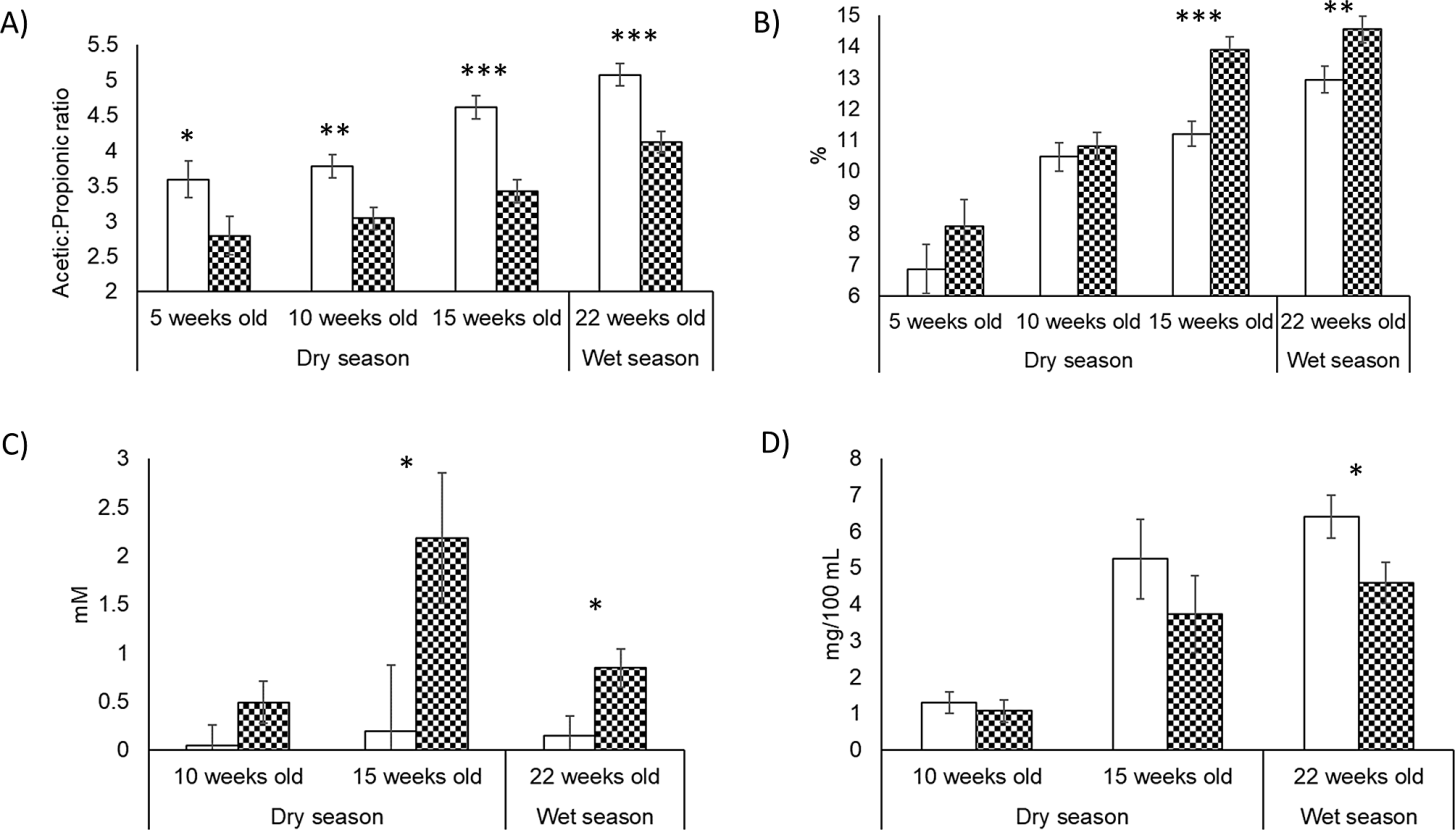
Ruminal fermentation metabolites (**A**) acetate: propionate ratio, (**B**) butyrate, (**C**) formate and (**D**) ammonia-N in C+ (▓) or C- (□) at 5, 10, 15 and 22 weeks old. Significant differences between 3-NOP and control (*P* < 0.001) ***, (*P* < 0.01) **, (*P* < 0.05) * for each time point

The treatment effects on rumen fermentation parameters and body weight in calves (pre-ruminant, suckling up to 5.5 months of age) from birth until weaning are shown in Fig. 3 and Supplementary Tables 6– 9. No differences were observed in body weight between the control and treated calves during the supplementation period (*P* = 0.291–0.668). Rumen VFAs showed increases (*P* < 0.05) in relative concentrations of propionate, butyrate, valerate and branched chain fatty acids, and a decrease (*P* < 0.05) in total VFA concentration (week 10 and 15), acetate and A: P ratio were observed in C + during the treatment period (Fig. 3A-B and Supplementary Tables 6– 9). Ammonia concentration significantly decreased in C + compared with C- at 22 weeks (*P* = 0.031) on treatment, which corresponds to the early wet season (Fig. 3D). Rumen organic acids were also significantly affected by 3-NOP treatment, with increases in rumen formate at 15 and 22 weeks (*P* = 0.044; 0.018) of age (Fig. 3C) and an increase in succinate (*P* = 0.050) and a decrease (*P* = 0.034) in fumarate at 15 weeks (Supplementary Table 8).

#### Post 3-NOP effect

The calves had access to their respective treatments (3-NOP or Control) from the first week of life until weaning (22 ± 2 weeks old). Due to the time differences in calving, the weaners (weaned young cattle up to 12 months of age) were assigned to two groups for the post-supplement analyses. Group 1 (28 weaners ± 1 week of age) were 5 weeks older than group 2 (17 weaners (± 2 weeks of age). Each group was weaned at the same age (22 weeks old).

**Fig. 4:**
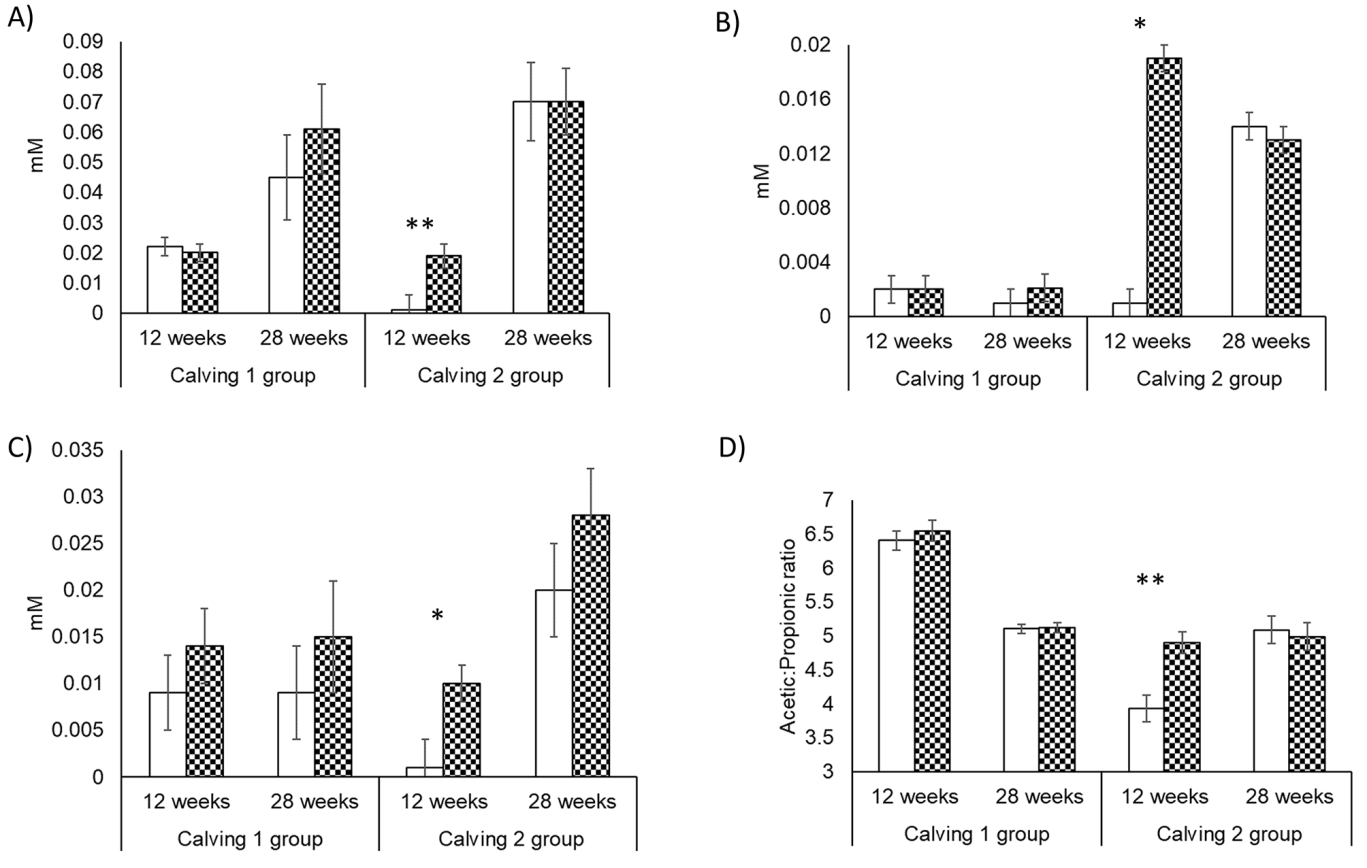
Ruminal fermentation metabolites formate (**A**), fumarate (**B**), lactate (**C**) and acetate: propionate ratio (**D**) in grazing weaners from calving 1 and calving 2 after 12- and 28-weeks post-treatment with 3-NOP (▓) or control (□). Significant differences between 3-NOP previously treated and control animals(*P* < 0.01) **, (*P* < 0.05) * for each time point and calving group

**Fig. 5:**
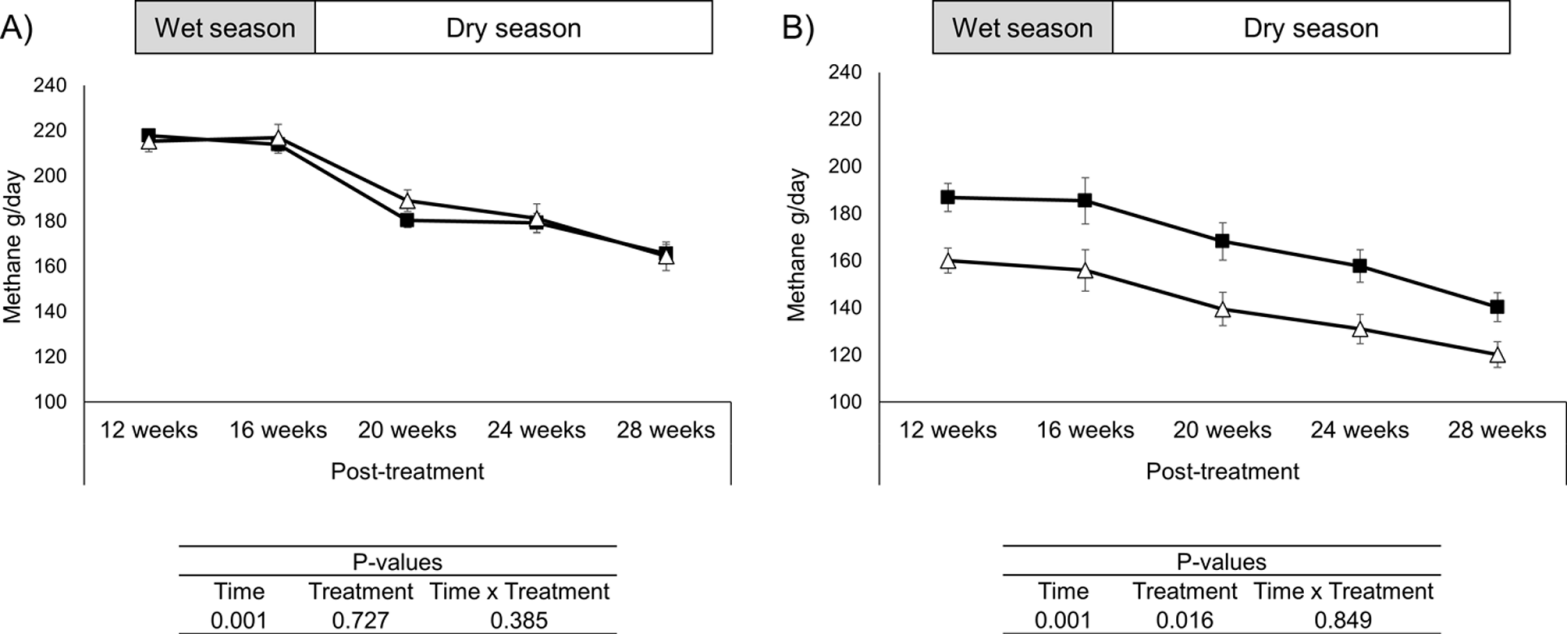
Methane (g/day) emissions in grazing weaners (calving group 1 (**A**) & calving group 2 (**B**)) from 12 to 28 weeks post-treatment with 3-NOP (Δ) or control (▀)

No difference (*P* > 0.05) in ruminal fermentation parameters (Fig. 4) and methane (*P* = 0.727) production (Fig. 5A) were found in the weaners from group 1 at 12 and 28-weeks post treatment (Supplementary Tables 10 and 12). For group 2 weaners, differences (*P* < 0.05) in ruminal fermentation parameters were detected at 12 weeks post treatment with decreases in total VFAs, propionate and valerate and increases in acetate, formate, lactate, fumarate and A: P ratio in C + weaners (Fig. 4 and Supplementary Table 11), with no differences observed at 28 weeks post treatment (Supplementary Table 13). Previously treated weaners (C+) in group 2 emitted less methane (*P* = 0.016), 10–15% less, than the control group (C-) for the 20 weeks that methane was measured (Fig. 5B) (from 8 to 28 weeks post treatment).

#### Dam effect in untreated offspring

The dam treatment (H- or H+) did not have any effect (*P* > 0.05) on the rumen fermentation parameters and body weight in the untreated suckling calves (C-), except for fumarate at week 15 (*P* = 0.015) and butyrate at week 22 (*P* = 0.015) (Supplementary Tables 14– 17). Also, the methane production and rumen metabolites in the untreated offspring were not affected (*P* < 0.05) by the dam treatment at 3- and 8-months post weaning (Supplementary Tables 18– 19).

### The rumen microbiota in offspring

#### 3-NOP effect

**Fig. 6:**
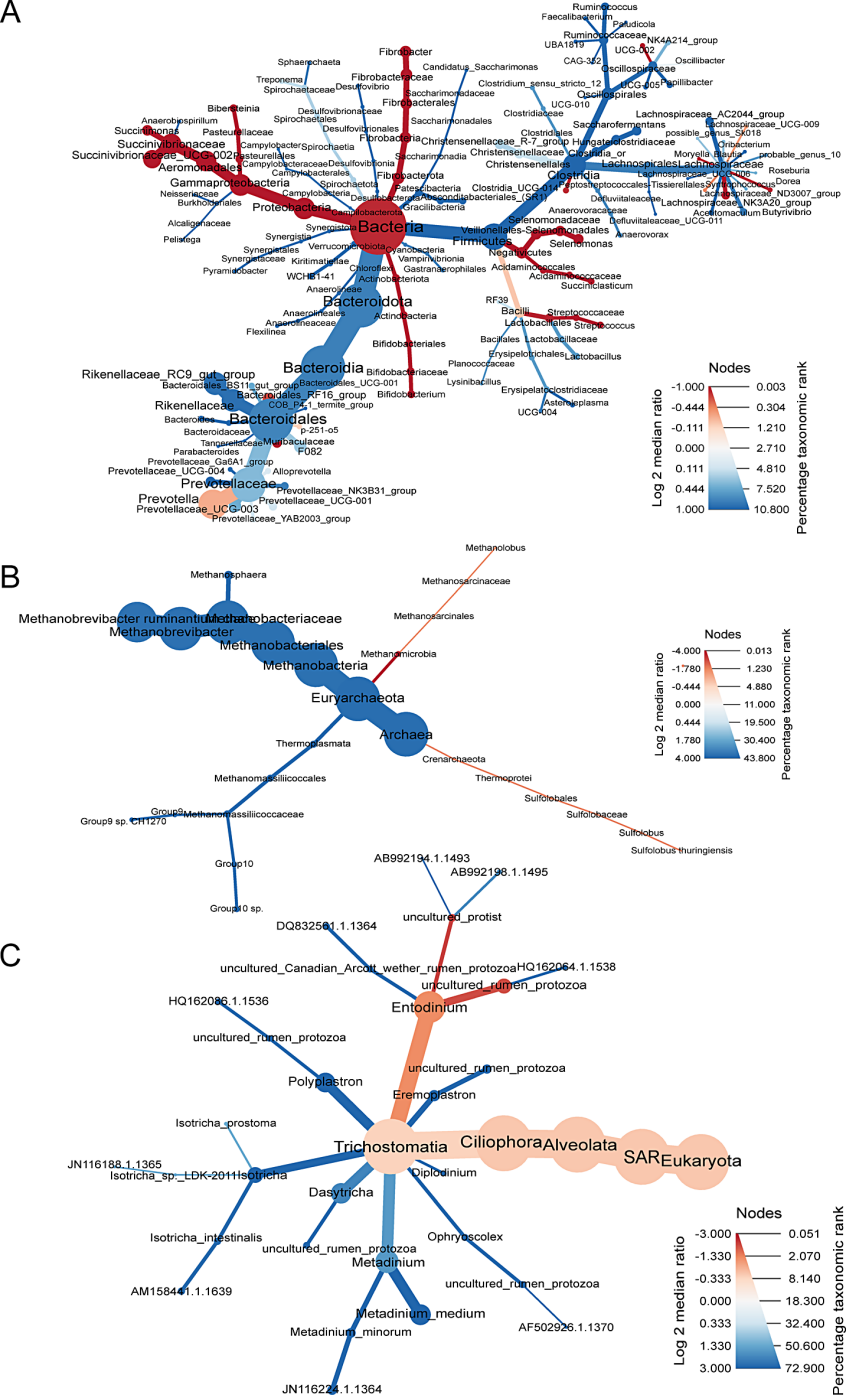
Differential taxonomic heat trees of rumen bacteria (**A**), archaea (**B**) and protozoa (**C**) communities selected by sPLS-DA showing log2 median ratio between suckling C+ (red) versus C- (blue) during the treatment period for a defined taxonomy. Node colour represents the log2 median ratio between the mean relative abundance of the taxa for each treatment, while the node size reflects the mean overall percentage of data assigned to the taxonomic rank

Using sparse PLS Discriminant Analysis (sPLS-DA), ASV’s that best characterised the calf treatment groups were determined [19]. Clustered image heatmaps for the selected rumen bacterial, archaeal and protozoa ASVs of calves at 5, 10, 15 and 22 weeks of age are shown in Supplementary files 4, 5 and 6 respectively (week 15 was excluded for archaea due to sequencing quality and protozoa were not detected in week 5). The selected ASVs based on the sPLS-DA analysis are visualised using a differential taxonomic heat treep (Fig. 6). The bacterial ASVs (Fig. 6A) positively associated with C + were classified to the families *Muribaculacaea* and *Succinivibrioacea*, and the genera *Selenomonas*, *Candidatus Saccharimonas*, *Succiniclasticum*, and *Fibrobacter*, while the families *Prevotellaceae*,* Christensenellaceae*,* Bacteroidales F082*, and *Rikenellaceae RC9* were positively associated with C-. Regarding the archaeal community structure (Fig. 6B), *Methanobrevibacter ruminantium*, *Methanomassiliicoccaceae family* and *Methanosphaera* spp were positively associated to C-. The protozoa ASVs (Fig. 6C) positively associated with C + treatment was classified to *Entodinium*, while *Metadinium*,* Dasytricha* and *Polypastron* were associated with C-.

Quantitative PCR analysis of the effect of 3-NOP on the abundance of rumen total methanogens (McrA), *Methanobrevibacter spp.* and *Methanomassiliicoccaceae* family in calves are shown in Supplementary Fig. 3. The methanogen abundance (McrA) decreased (3–6 folds) during the treatment period for C + compared with C-, except at 15 and 22 weeks old. The *Methanomassiliicoccaceae* family was significantly decreased (3–6 folds) at 5, 10, 15 and 22 weeks of age, while *Methanobrevibacter* was decreased (1-6-fold) at 5, 10 and 15 weeks of age for C+.

#### Post 3-NOP effect

Using sparse PLS Discriminant Analysis (sPLS-DA), ASV’s that best characterised the weaner treatment groups post treatment were determined [19]. Clustered image heatmaps for the selected rumen bacterial, archaeal and protozoa ASVs of weaners at 12 and 28 weeks post-supplementation are shown in Supplementary files 4, 5 and 6 respectively. The Bacterial ASVs positively associated with 3-NOP during the post-treatment period at 12 and 28 weeks post-supplementation (Supplementary file 4, weeks 35 & 55) were classified mainly to the families *Prevotellaceae*,* Bacteroidales F082* and *Christensenellaceae*, and genus *Succiniclasticum spp*.; while the phylum Verrucomicrobiota, the families *Bacteroidales RF16* and *Lachnospiraceae* and genus *Fibrobacter* were associated to the un-treated animals. Archaeal ASVs (Supplementary file 5, weeks 35 & 55) classified to *Methanobrevibacter ruminantium.* and *Methanomassiliicoccaceae* family were positively associated with un-treated weaners while *Methanosphaera spp.*, *Methanobrevibacter gottschalkii* and *smithii* were associated with previously treated weaners. The protozoa ASVs (Supplementary file 6, weeks 35 & 55) positively associated with the 3-NOP treatment during the post-supplement period were classified to *Entodinium*, *Diplodinium* and *Eudiplodinium*, while *Metadinium*,* Dasytricha* and *Isotricha* were associated with the untreated animals.

Quantitative PCR analysis of the total methanogens (McrA), *Methanobrevibacter spp.* and *Methanomassiliicoccaceae* family abundances at 12 and 28 weeks post treatment (data not shown), showed no significant differences between the experimental groups.

#### Dam effect in untreated offspring

The clustered image heatmaps for the rumen bacterial, archaeal and protozoa ASVs associated with the dam effect on untreated animals while suckling and post-weaning are shown in Supplementary files 7, 8 and 9 respectively.

Bacterial ASVs (Supplementary file 7, weeks 5, 10, 15 and 22) of untreated suckling calves from 3-NOP treated dams (H+) were associated with the phylum *Verrucomicrobiota*, families *Prevotellaceae*,* Rikenenllaceae_RC9*,* Bacteroidales RF16*,* BS11 and Muribaculacaea*, and genera *Candidatus Saccharimonas* and *Ruminococcus.* Archaeal ASVs in the C- suckling calves (Supplementary file 8, weeks 5, 10 and 22) which assigned to *Methanobrevibacter* and *Methanosphaera* spp were positively associated with treated dams (H+) and *Methanomassiliicoccaceae family* to the untreated dams. The dam effect on the rumen protozoa structure of their offspring (C-) while suckling is shown in Supplementary file 9 (weeks 10, 15 and 22). ASVs assigned to *Dasytricha* were positively associated with the treated dams, while *Polyplastron and Isotrycha* were associated with the un-treated dams.

The dam effect persisted in the offspring at 12 and 28 weeks post weaning, with ASVs classified to the families *Prevotellaceae*, *Rikenenllaceae_RC9*, *Christensenellaceae* and genera *Fibrobacter*, *Ruminococcus* and *Succinoclasticum* positively associated to the weaners from the treated dams (Supplementary file 7, weeks 35 and 55). Archaeal ASVs (Supplementary file 8, weeks 35 and 55) assigned to *Methanobrevibacter* and *Methanosphaera* spp being positively associated to the weaners from untreated dams, while the *Methanomassiliicoccaceae family* with the weaners from treated dams. Protozoa (Supplementary file 9, weeks 35 and 55) ASVs assigned to *Eudiplodium*,* Diplodinium*,* Ophryoscolex* and *Eremoplastron* in weaners from treated dams, while *Dasytricha*,* Polyplastron* and *Entodinium* were positively associated with untreated dams.

#### Calf microbiome development

**Fig. 7:**
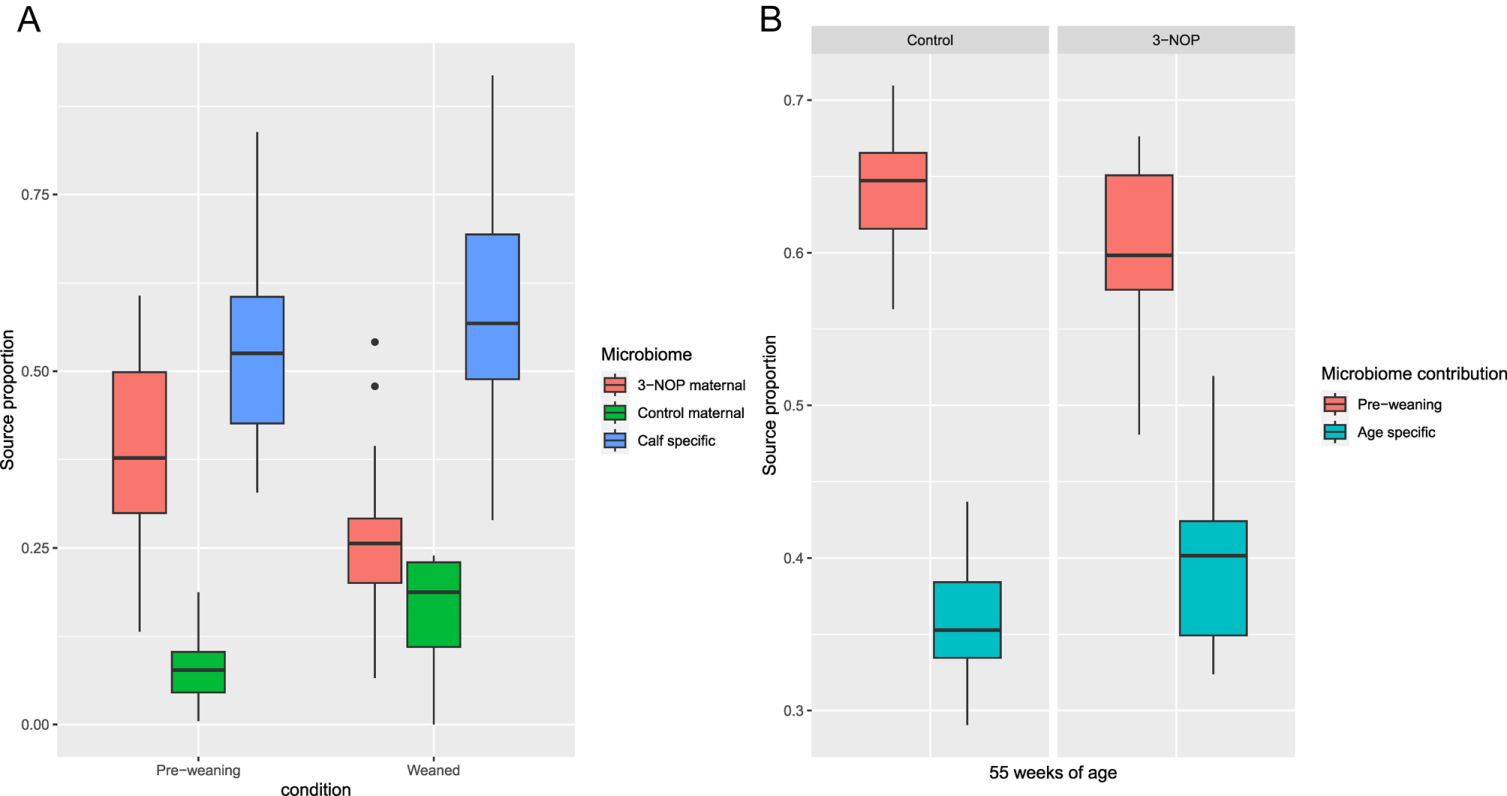
FEAST estimation of source microbiome contribution to sink microbiome. (**A**) Control calf microbiomes at pre and post weaning, sources: control and 3-NOP dams. (**B**) Control and 3-NOP post treated yearling animals, sources: pre-weaning calves. Box plots indicate the median (central lines), IQR (hinges) and the 5th and 95th percentiles (whiskers)

Source tracking, using fast expectation-maximization microbial source tracking (FEAST) found that in pre-weaned control animals, 47.2% of the calf microbiome was attributed to the adult microbiomes while 52.8% was unknown representing a calf specific microbiome. Of the adult identified microbiome, 39.5% resembled the microbiome of a 3-NOP treated dam and 7.7% resembled the control dam microbiome (Fig. 7A). In weaned animals 41.8% of the calf microbiome were linked to the adult microbiome with 25.9% resembling the microbiome of a 3-NOP treated dam and 15.9% resembling the control dam microbiome.

Furthermore, the microbiome proportions of the yearling animals at 55 weeks of age were attributed to be 64.1 and 60.5% resembling those of the animal pre-weaning for the control and 3-NOP animals respectively and with 35.9 and 39.5% as unknown or age specific (Fig. 7B).

## Discussion

The process of microbial colonisation of the gut is receiving much attention in both human medicine and animal agriculture due to the importance of these organisms to the health and nutrition of the host [5, 7]. The transfer of microorganisms from the dam to offspring, host specific characteristics of the gut, and diet of the juvenile animal all interact to influence the structure and function of the microbiome. The non-secretory forestomach of the ruminant matures from a rudimentary organ during early suckling to a fully functional rumen by weaning, which provides a unique model to study the factors that govern the process of microbial colonisation in the neonate [20]. In this study we established distinctive microbial profiles by perturbing the rumen microbiota with the anti-methanogenic compound 3-NOP to assess the dam effect, and nutritional intervention of the juvenile offspring on microbial structure and function of rumen up to 12 months of age. Characteristic bacterial, archaeal and protozoal populations were found to be common to both dams and calves treated with 3-NOP. A small subset of these microbes was also noted to be positively associated with untreated offspring from treated dams which might indicate a link between maternal gut microbiota and a profile of organisms that establish in the juvenile during the suckling period. However, treatment of suckling calves with 3-NOP produced a distinctive microbiota pattern which endured for some animals more than 6 months after treatment was withdrawn at weaning.

Although a small proportion of the calf rumen microbial structure was attributed to a dam effect, no significant phenotypic differences in rumen fermentation profiles or methane production levels were observed in the un-treated offspring from the two maternal groups. The dietary intervention with 3-NOP applied to the juvenile animal, within the second calving group however produced distinctive rumen fermentation profiles which continued for several months after weaning, along with lower methane emissions until at least 28 weeks after treatment was withdrawn (52 weeks of age). Previous studies have reported similar results under more controlled feeding conditions. Meale et al., [13], observed a persistent reduction in methane and a distinctive rumen microbial structure in dairy cattle till at least 60 weeks of age, when the animals were dosed daily by oral gavage with 3-NOP for 12 weeks after birth. Abecia et al. [10, 12] showed similar responses in young goats up to 4 months post-treatment, when kids and does were both treated with the anti-methanogenic compound, bromochloromethane, during the first 2 months of lactation before weaning. These previous studies and the current study conclude a reduction in methane (10–15%) and alteration to the microbiota is sustained for several months after the ceasing of daily treatment of the suckling animal with an anti-methanogenic compound, even though the fermentation profile may revert to that of untreated animals.

### Effects of 3-NOP on rumen fermentation and microbiota during treatment

During the supplementation period, 3-NOP treated animals (dams and calves) showed a shift in fermentation from acetate to volatile fatty acids that were longer in length, particularly propionate that is a major gluconeogenic precursor in ruminants [21]. This pattern of fermentation along with an increase in branched chain fatty acids, has been reported previously in studies using 3-NOP [22–25] or other methane inhibitors such as halogenated methane analogues [26–28]. Rumen formate was also significantly higher in 3-NOP dams and calves during the treatment period, which is in line with previous published studies using methane inhibitors [26, 29, 30]. A meta-analysis also identified that an increase in formate could be a response to methane inhibition in ruminants [31] and might help to maintain a low partial pressure of H_2_ in the rumen fluid [32]. As a consequence, an increase in formate in the rumen may prove a useful biomarker and indicator of successful methane inhibition in future studies.

Similar shifts in microbial populations occurred in both dams and calves treated with 3-NOP, which appeared consistent with the mode of action of the compound (direct inhibition of methanogenesis), the rumen metabolic changes detected and a likely increase in hydrogen levels as observed in previous studies using 3-NOP in intensive farming systems [18, 25, 33]. These changes were associated with lower abundances of rumen methanogens and changes to their community structure, highlighted by a reduction in the relative abundance of *Methanobrevibacter ruminantium* (hydrogenotrophic) and *Methanomassiliicoccaceae family* (methylotrophic). This was a similar response to a previous report for cattle fed a roughage diet with both hydrogenotrophic and methylotrophic methanogens being negatively impacted by 3-NOP and a concurrent shift in the community structure of both populations [17]. However, Meale et al., [13] did not find a negative effect of 3-NOP on methylotrophic methanogens in young dairy calves, a finding which might reflect the higher quality diet and the subsequent overall higher proportion of methylotrophic species. The 3-NOP effect on the rumen bacterial structure in supplemented animals was positively associated with propionate producers in both the treated dams and calves, with increases in *Succiniclasticum spp* and *Candidatus Saccharimonas* observed. *Succiniclasticum spp* can convert succinate to propionate in the rumen [34], while *C. Saccharimonas* has been positively associated with propionate increases in dairy cows [35]. Some bacterial populations were only positively associated with 3-NOP treatment in either dams or calves, with *Prevotellaceae* associated in dams only, and *Fibrobacter* and *Succinivibrioacea* in the calves. These differences are likely to be influenced by the strong effect of diet on the establishment of the rumen microbial community and the age-related changes (from pre-ruminant to ruminant) that occurred in the maturing offspring [10, 20]. *Succinivibrioacea* species generally are observed in early life prior to transitioning to higher fibre diets and tend to exhibit an inverse relationship to methanogen abundance or inhibition [26, 36, 37]. Previously, *Fibrobacter* was also positively associated with 3-NOP treated dairy calves [13] and species, such as *F. succinogenes*, are not susceptible to H_2_ accumulation in the rumen [27]. The *Prevotellaceae* family has the highest representation of genes for the propionate pathway [38] and has also been associated with 3-NOP treatment in previous studies [17]. The *Rikenellaceae*, and the *Bacteroidetes F082* group have previously been reported as significant bacteria in cattle grazing tropical pastures in Australia during the dry season [39]. Their relative reduction in the treated calves may indicate a greater sensitivity to H_2_ concentration.

Ruminal ciliate protozoa are indirectly involved in rumen methane production, through their production of H_2_ and symbiotic association with methanogens [40]. Entodiniomorphid protozoa have been linked to lower methane production in sheep compared to holotrycha [41], and some holotrycha species have been identified as greater H_2_ producers [42]. Similar relationships were observed in our study, with the treatment (low methane) positively associated with entodiniomorphs, particularly *Entodinium*. This effect was not observed by Meale and co-workers [13], in young dairy-calves treated with 3-NOP, which might be explained by the late rumen colonization by protozoa in that study (protozoa only visible at ≥ 22 weeks), as the calves were separated from their mothers at birth. Hydrogenosomes which attract methanogens to associate with protozoa have been identified in species of Isotricha and Dasytricha, but they were not found in *Entodinium caudatum* [43, 44]. The positive association of 3-NOP treatment with *Entodinium* may arise from a lesser dependence on the methanogen population than other protozoa.

### Effects of 3-NOP on rumen fermentation, methane production and microbiota post-treatment

The group of weaners that were born later in the study (calving 2) showed significant ongoing effects on rumen metabolites and methane emissions unlike the earlier group (calving 1). In addition to the ~ 15% lower methane emissions, a shift towards acetate and an increase in formate, succinate and lactate was found in the same group of animals at 12 weeks post-treatment. The increase in acetate was unexpected, but the other shifts in metabolites observed are frequently associated with a reduction in methane and add support to the conclusion that methane emissions in these animals were lower than the control group. Microbial metabolic processes, such as reductive acetogenesis, propionogenesis, formate formation and an increase of microbial biomass production when CH_4_ is inhibited, have been identified as nutritionally useful [H] sinks in the rumen which may have contributed to maintaining a reduction in methanogenesis in the calving 2 group [21, 31, 32, 45]. We are unable to explain why the second calving group performed differently, but hypothesise consumption of the 3-NOP mixture may have commenced earlier in group 2 due to social learning from the older calves. Although it has been reported that the presence of older animals influences feeding behaviour before and after weaning in dairy calves [46], further studies monitoring the consumption in the young animals should be carried out to confirm our hypothesis.

Some of the microbial changes observed during the treatment period persisted in the weaners up to 28 weeks after the supplementation ceased, however the effect was diminished once 3-NOP was withdrawn. The *Methanomassiliicoccaceae* family and *Methanobrevibacter ruminantium* were still negatively impacted, while the bacteria *Christensenellaceae*,* Succinuclasticum*,* Prevotellaceae* and some entodiniomorphs protozoa where still positively associated with the weaners that received the treatment earlier in life. Similar long-lasting effects to the rumen microbial structure of mature animals that received an intervention during the pre-weaning phase have previously been reported, which highlights the importance of early life programming and its impact on the mature animal [8, 10, 13, 47].

### Dam effect on untreated offspring

There is strong interest in understanding the maternal influence on establishment of the rumen microbiota in their offspring, and its implications in the health and metabolism of the mature animal [5–7]. Even though the microbial composition was primarily modulated by the diet and age of the host in our study, a small distinct microbial signature associated with the dams was observed during suckling and post-weaning in the offspring. Microbial groups, characteristic of 3-NOP treated dams, were identified in the untreated calves and weaners of the treated dams, such as bacteria assigned to *Prevotellaceae*,* Christensenellaceae*, *Candidatus Saccharimonas* and *Succinoclasticum*. Interestingly, the archaeal and protozoal community structure in weaners showed an opposite effect to that observed during the suckling phase, with the *Methanomassiliicoccaceae* family and some entodiniomorph protozoa being positively associated with weaners from treated dams, which might indicate that populations were equilibrating during the pre-ruminant phase [48]. However, the small microbial differences observed did not translate into significant changes in methane production or rumen fermentation metabolites in the offspring from treated dams. Despite the lack of effect in these parameters, dam effect should be further studied and considered as a strategy for early life programming, as it may have a significant impact when targeting other desirable phenotypes, such as feed efficiency, health parameters or diet transitioning.

In the current study, the interaction between the calf and dam treatments could not be assessed due to the lack of effect on methane emissions in the first calving group and the low numbers of animals in the second group. However, some studies reported greater methane reduction in small ruminants when both the mother and offspring were treated with an anti-methanogenic compound until weaning [10, 12].

### Calf microbiome development

Further to this, it was observed that ~ 50% of the pre-weaning calf bacterial microbiome resembled that of the adult dam and that later, the pre-weaning microbiome comprised ~ 65% of the yearling animal. This is similar to other studies that indicate that early life colonisation of the rumen is by a core successional microbiome that then persists through to later life [36]. Interestingly, a greater proportion of the control pre-weaned animal microbiome resembled that of a 3-NOP treated dam. The utility of using FEAST allows to not only track the potential source contribution but also can be used as a similarity metric for microbiome characteristics. This higher resemblance of the young calf microbiome to a low methane adult (3-NOP treated) reflects the natural state of the young rumen and lower methane levels. Together these observations suggest that reinforcing the young low methane rumen through supplementation of 3-NOP in early life may impact the core successional microbiome that is retained post-treatment, but the longevity of the effect and functional changes needs further investigation.

## Conclusion

In conclusion, this study provides evidence that treating calves with 3-NOP soon after birth through till weaning can sustain a reduction in methane emissions for up to seven months after the treatment is withdrawn but further studies are required to determine the consistency, duration of the response in the maturing animal, optimal route of administration, timing of intervention and the adequate dosage of 3-NOP.

Supplementation of 3-NOP was successfully delivered in grazing conditions under voluntary intake of the compound, leading to the detection of differences between the treated and untreated animals similar to the studies under more controlled feeding conditions. Early life intervention had an enduring impact on the rumen microbial structure of young animals up to 28 weeks post weaning. Our results suggest that early life colonisation of the rumen is by a core successional microbiome that then persists through to later life, with a higher resemblance of the young calf microbiome to a low methane emitting adult. Therefore, programming the rumen early in life might be used to target other desirable phenotypes in the adult animal for particular farming systems.

## Methods

### Experimental design and sampling

Forty-eight pregnant heifers (*Bos indicus*) were selected from a larger cohort on estimated age of pregnancy and randomly allocated to two groups: One group of twenty-four heifers (537 ± 17 kg) received the anti-methanogenic compound 3-NOP (H+) for approximately two months prior to calving and six months after calving, and the other group of 24 animals (538 ± 16 kg) received a control treatment (H-). The respective treatment groups were held in two separate paddocks containing the same tropical pasture. Due to uncontrolled natural events (illnesses and calf losses during lactation period) the final number of heifers per group that completed the trial were 21 H- and 24 H + with their respective calves.

Six weeks prior to calving, each group of heifers (H) were further divided into two groups and were held in four separate paddocks containing the same tropical pasture (12 pregnant heifers per paddock). The calves (C, pre-ruminant, suckling until 22 ± 2 weeks of age)) subsequently born in each group were treated with control (-) or 3-NOP (+) until weaning (22 ± 2 weeks of age), resulting in four experimental groups: H+/C+, H+/C-, H-/C + and H-/C-. A custom enclosed area was designed to allow the calves access to their treatment while excluding the heifers from the calves’ treatment. Furthermore, the feed troughs which contained the treatment for the dams, were not accessible by the calves. The calves received the treatment (control or 3-NOP) from the first week of life until weaning. After weaning, all the weaners (W, weaned young cattle up to 12 months of age) were grazed together in the same paddock to monitor liveweight gain, rumen fermentation and methane production post treatment (until 53 ± 2 weeks of age). (Trial timeline, Supplementary Fig. 1).

Rumen fluid samples were collected, and body weights measured from all heifers at pre-treatment (Baseline), 6 weeks on treatment (pre-partum), 18 weeks on treatment (lactating), 23 weeks on treatment (lactating) and 30 weeks on treatment (lactating). Rumen fluid samples and body weight were collected from offspring (calves and weaner stage) at 5, 10, 15, 22, 35 and 55 weeks of age. Rumen fluid was collected by oesophageal intubation at 3 (± 1) h post offering the treatments. Samples were immediately frozen using dry ice and stored at − 80 °C until analyzed for ruminal fermentation metabolites and microbial community composition.

Sample material collected at 5 weeks of age was not sufficient for performing organic acids and ammonia analyses, therefore the DNA extraction for microbial characterisation and VFA analysis were prioritised. From 10 weeks of age, enough material was collected for performing all the analyses.

Due to the time differences in calving, two calving groups were considered for analytical purposes. Calves from the 1st calving group were 5 (± 1) weeks older than the 2nd calving group. 28 calves (± 1-week old difference) were classified as 1st calving group; and 17 calves (± 2 weeks old) were assigned to the 2nd calving group. Calves from the second calving group were sampled and weaned 4–5 weeks after animals from calving 1 due to the calving time difference.

### Paddocks and pasture composition

The groups of animals were held grazing in separated replicated paddocks at different stages during the trial. The paddocks were replicates with similar pasture composition and size (15 ha each). Paddocks grasses and legumes composition: *Urochloa sp*, Rhodes Grass (*Chloris gayana*), Bluegrass (*Dichanthium sericium*), Buffel (*Cenchrus ciliaris*) & Spear grass (*Heteropogon contortus*); legumes: Seca Stylo (*Stylosanthes scabra*), Verano (*Stylosanthes hamata*) & Desmanthus (*Desmanthus sp*) Table 1.

**Table 1:** Paddock pasture nutrient composition

	Dry Season	Wet season
Total Crude Protein, g/kg DM	37	69
NDF, g/kg DM	767	731
ADF, g/kg DM	445	413
Hemicellulose, g/kg DM	318	320
Organic Matter, g/kg DM	943	910
Ash, g/kg DM	57	90
ME MJ/kg DM	6.12	6.93

### Treatments formulation and dosage

The treatment used in the trial were: Control: propane-1,2-diol adsorbed on silicon dioxide; and 3-NOP: 3-nitroxypropanol diluted in propane-1,2-diol adsorbed on silicon dioxide (3-nitroxypropanol concentration 10.9%. Lot No. UQ70384011).

The treatments (Control or 3-NOP; 3.71%) were mixed with steam flaked barley (88.9%) and molasses (7.4%). The three ingredients were mixed for four minutes using a feed mixer. The treatment mixes (Control or 3-NOP + Barley + Molasses) were prepared separately every 10 days and stored in a cold room until offered to the animals. Samples from 3-NOP and each final mix (3-NOP + Barley + Molasses and Control + Barley + Molasses) were collected periodically and shipped to DSM Nutritional Products (Global R&D Analytics – NIC-RD/A; 4002 Basel; Switzerland) to confirm the 3-NOP target concentration for each 3-NOP batch during the trial.

Each group of heifers was offered 675 g/head/day (divided in two offers: 8:00 and 14:00 h) of their respective treatment (3-NOP mix or Control mix), which was equivalent to 2.5 g of 3-nitrooxypropanol (3-NOP) per animal/day. The dose was based on previous in-vivo trial [17] which reduced methane by approx. 38% in *Bos indicus* cattle fed tropical hay. The heifers received the treatments in long feed troughs located in an enclosed area at the paddocks water points. All animals were able to access the supplement at the same time and the treatment mix was evenly distributed in the troughs to limit variation in intake between animals. Heifers received the treatment from 9 ± 2 weeks before calving, until their calves were weaned (22 ± 2 weeks in lactation).

The calves had access to their respective treatments (3-NOP or Control) from the first week of life until weaning (22 ± 2 weeks old). The target dose of active ingredient per calve was 3–6 mg of 3-nitroxypropanol /kg BW, based on previous research [13]. During the first 3 ± 1 weeks of life, the calves received the treatments mixed with molasses [750 g of molasses/group/day]. From the third week (± 1 week) of life calves received the treatments mixed with barley (90%) and molasses (7.7%) + 3-NOP or Control, divided in 2 offers, 8:00 and 14:00 h. The amount of the treatment mix offered was adjusted monthly to achieve a target dose equivalent to 3–6 mg of 3-nitroxypropanol/kg BW (calves weighed monthly). The objective of the study was to simulate practical grazing conditions; therefore the supplements were offered ad libitum to the animals as a group and the individual intakes per animal would have varied during the trial.

### Gas measurements

During the post-treatment period, weaners were grazed together in a 35-ha paddock and were introduced to Greenfeed Emission Monitors (GEMs) (C-Lock Inc., Rapid City, SD, USA) [49, 50]. Two GEM units were placed in the paddock adjacent to water and to each other, to measure daily enteric methane emissions for 6 months. To control the number and duration of methane measurements, pellets were provided to each animal with a maximum of 5 feeding sessions/d and a minimum of 4.5 h between sessions. In each feeding session the maximum quantity of pellets delivered per animal was 240 g (4 drops of approximately 60 g with 45s interval between drops). If cattle did not remain to receive the 4 drops in 1 visit, they could make further visits to the GEM in that session until all pellets drops were dispensed. For emission data to be recorded, animals were required to have their head in the unit for at least 2 min as detected by a proximity sensor. Air filters on the GEMs were changed weekly and gas sensors were calibrated automatically each day at 0430 h when no cattle were accessing the units. Daily emission estimates (g CH_4_/d) were all calculated manually using the data provided by C-Lock to generate emission estimates for individual animals on individual days.

### Chemical analysis

Concentrations of VFAs (acetate, propionate, n-butyrate, iso-butyrate, iso-valerate and n-valerate) were measured by gas chromatography (GC) as described by Gagen et al. [51]. Iso-valerate (3-methyl butyrate) includes 2-methyl butyrate, which co-elutes.

The NH_3_-N concentration was determined by a colorimetric method following Chaney and Marbach [52].

An UltiMate^®^ 3000 HPLC system (Dionex, Sunnyvale, CA, USA) with a dedicated Photodiode Array Detector and an Autosampler was used to determine the presence of organic acids in rumen samples supernatants as described by Gagen et al., [51].

Forage samples were dried in a forced-air oven at 105 ºC prior to grinding. Feed samples were ground through a 1 mm sieve using a Tecator Cyclotec 1093 (FOSS, Hillerød, North Zealand, Denmark). DM, ash, NDF, ADF, hemicellulose, organic matter, gross energy and total nitrogen contents were analysed at the CSIRO Floreat laboratory (Floreat, WA, Australia). The nitrogen values were converted to CP by a factor of 6.25.

### DNA Extractions and Illumina MiSeq Sequencing analyses

DNA extractions from rumen samples were performed on a 1 ml sample as described by Martinez-Fernandez et al. [26]. PCR amplification of the 16 S rRNA and 18 S rRNA genes were used to characterize the rumen microbial populations. PCR amplification targeting the bacterial v4 region used the primers F515 (GTGCCAGCMGCCGCGGTAA) and R806 (GGACTACHVGGGTWTCTAAT) [53]. Archaeal 16 S rRNA sequences were amplified targeting the v6-v8 region using Ar915aF (AGGAATTGGCGGGGGAGCAC) [54] (reverse complement of the originally described forward archaeal primer 0915a [55]) and Arch r1386 (GCGGTGTGTGCAAGGAGC) [15]. Protozoal 18 S rRNA sequences were amplified using the primer pairs GIC1050F (GGGGRAACTTACCAGGTCC) and GIC1578R (GTGATRWGRTTTACTTRT) [56]. Each DNA sample was amplified using target specific primers and then tagged with unique dual barcodes and the index 1 (i7) and index 2 (i5) AmpliSeq adapters (www.illumina.com). Amplification included an initial denaturation step at 94^o^C for 1 min and then either 25 cycles for bacteria or 30 cycles for archaeal and protozoal of 94^o^C for 15 s, 55^o^C for 30 s and 72^o^C for 45 s, followed by a final step of 72^o^C for 7 min. Amplification products were visualized by performing gel electrophoresis. Product quantities were calculated, and an equal molar amount of each target product was pooled. The pooled target products were run in a 1.5% agarose gel and bands were visualized and excised under blue light trans-illumination. The amplicons were gel purified with a QIAquick Gel Extraction Kit (Qiagen, Hilden, Germany) prior to submission for 2 × 250 bp Illumina MiSeq sequencing (Macrogen Inc., Seoul, South Korea).

Paired-end short-read sequence data generated on the Illumina MiSeq was processed using the following steps. Amplification primers were removed from sequences using cutadapt [57] and sequences less than 200 bp after adaptor removal were discarded. Sequencers were then processed using the VSEARCH package [58]. Firstly, fastq sequences were filtered by truncating to 200 bp and discarding reads with an expected error above 1.0. Further quality filtering of fastq sequences included denoising and chimera detection using the cluster_unoise and uchime3_denovo commands from VSEARCH which implement UNOISE and UCHIME algorithms respectively [59, 60]. Clustering of sequences to Amplicon sequence variants (ASVs) [61] was performed using usearch_global option in VSEARCH based on the original USEARCH algorithm of Edgar [62]. Taxonomic classification of ASVs was done using the assignTaxonomy command implemented in the DADA2 R package [63]. Bacterial taxonomy utilised the DADA2 formatted reference database Silva version 138.1 training set (https://zenodo.org/records/4587955), archaeal taxonomy utilised the RIM-DB database [64] and protozoal taxonomy was referenced to Silva Eukaryotic 18 S, v132 (https://zenodo.org/records/1447330). Further analysis of microbiota diversity and identification of ASVs significantly altered by supplementation or dam was performed in R following the compositional data analysis [65], using packages mixOmics [66], Phyloseq [67], vegan [68], and Metacoder [69]. ASV tables were pre-filtered to remove low count ASVs across samples at a cutoff of 0.01% of the total count, then an offset of 1 was added to the ASV count table to account for zeros prior to cantered log transformation. Using the mixMC framework for multivariate analysis in mixOmics, we initialled produced a PLS-DA model and then tuned and evaluated the models performance with respect to treatment groups. The number of ASV features used for each component was then determined through improvements in the error model resulting in features that that were used to produce a final sPLS-DA model.

Source tracking analysis of the control calf microbiomes was performed by fast expectation-maximization for microbial source tracking (FEAST) [70] using the combined ASV data from the control calves and their mothers at a given sampling point. Dam microbiomes were defined as either control or 3-NOP sources respectively and the control calves were defined as sinks. The FEAST results from each sample point defining the source contributions to each calf were combined into a single table and summary boxplots were generated for pre-weaning and weaned calves. Source tracking analysis of yearling animals’ microbiome was performed using the combined ASV data from all animals from birth to 12 months of age. Yearling animals were flagged as sinks, and their respective pre-weaning calf’s microbiome as source.

### Quantitative PCR analysis

The DNA samples were used as templates for quantifying the abundance of the mcrA gene for total methanogens, and the 16 S rRNA for *Methanobrevibacter* genus and *Methanomassiliicoccaceae* family. The primers and assay conditions used were previously published by Denman et al., [71] and Huang et al., [72]. Quantitative PCR (qPCR) analyses were run in quadruplicate from one DNA extraction on an Applied BiosystemsTM ViiATM 7 Real-Time PCR System (Thermo Fisher Scientific Inc.). Assays were set up using the SensiFAST SYBR^®^ Lo-ROX reagents (Bioline). Optimization of assay conditions was performed for primer, template DNA and MgCl_2_ concentrations. An optimal primer concentration of 400 nM, with a final MgCl_2_ concentration of 3 mM and DNA template concentration of 50 ng were used for each assay under the following cycle conditions: one cycle of 50 °C for 10 s and 95 °C for 2 min 30 s for initial denaturation, 40 cycles at 95 °C for 15 s and 60 °C for 1 min for primer annealing and product elongation. Fluorescence detection was performed at the end of each annealing and extension step. Amplicon specificity was performed via dissociation curve analysis of PCR end-products by raising the temperature at a rate of 0.05 °C/s from 60 to 95 °C. Changes in targeted populations were calculated using a relative quantification calculation and the 2 − ΔΔCt method, with the control group used as the calibrator and total bacterial ct (cycle threshold) values used as the reference value [73, 74].

### Statistical analyses

Data from heifers (dams) and calves were analysed as a univariate model using the GLM procedure of SPSS (IBM Corp., version 21.0, Armonk, NY, USA), with the animal as the experimental unit. The direct effect of 3-NOP treatment on dams and calves, or indirect effect of dam treatment with 3-NOP on offspring (for the dam effect only untreated offspring were included), was analysed for body weight, rumen fermentation metabolites and qPCR data. Effects post treatment of weaners with 3-NOP was analysed for CH_4_, body weight, rumen fermentation metabolites and qPCR data. Differences among means were tested using least significant difference (LSD) comparison test. Methane data from the weaners was analysed separately as a repeated-measures analysis using the GLM procedure of SPSS (IBM Corp., version 21.0, Armonk, NY, USA), with the animal as the experimental unit to study the post-treatment effect.

## Electronic supplementary material

Below is the link to the electronic supplementary material.

Supplementary Material 1

Supplementary Material 2

Supplementary Material 3

Supplementary Material 4

Supplementary Material 5

Supplementary Material 6

Supplementary Material 7

Supplementary Material 8

Supplementary Material 9

Supplementary Material 10

## Data Availability

The sequences generated in this study have been deposited in the European Nucleotide Archive under the accession number PRJEB68136 and all the metadata is presented in the main manuscript and additional supporting files.
