# Supplementary material for: Programming rumen microbiome development in calves with the anti-methanogenic compound 3-NOP
Source: Anim Microbiome. 2024 Oct 25;6:60. doi: 10.1186/s42523-024-00343-2 (PMC11515290; doi:10.1186/s42523-024-00343-2)
Supplement: Supplementary file 10 — Supplementary Material 10 [file 42523_2024_343_MOESM10_ESM.docx]

**Supplementary Table 1.** Rumen fermentation parameters and body weight in pregnant heifers grazing tropical forage (early dry season). Group comparison pre-treatment.

|  | H- | Pre-treatment | SEM | P-value |
| --- | --- | --- | --- | --- |
| BW kg | 537 | 538 | 11.9 | 0.954 |
| Rumen pH | 7.29 | 7.18 | 0.04 | 0.233 |
| Formate mM | 0.015 | 0.022 | 0.01 | 0.493 |
| Lactate mM | 0.082 | 0.092 | 0.02 | 0.816 |
| Succinate mM | 0.65 | 0.63 | 0.05 | 0.777 |
| Fumarate mM | 0.0014 | 0.0004 | 0.00 | 0.106 |
| Ammonia-N mg /100 mL | 5.72 | 6.33 | 0.31 | 0.335 |
| Total VFA mM | 72.3 | 71.7 | 2.89 | 0.914 |
| acetate % | 73.3 | 73.8 | 0.21 | 0.182 |
| propionate % | 13.8 | 13.3 | 0.15 | 0.112 |
| iso-butyrate % | 0.74 | 0.84 | 0.04 | 0.229 |
| n-butyrate % | 10.21 | 9.98 | 0.13 | 0.378 |
| iso-valerate % | 0.96 | 1.03 | 0.04 | 0.434 |
| n-valerate % | 0.75 | 0.73 | 0.01 | 0.628 |
| n-caproate % | 0.30 | 0.31 | 0.01 | 0.752 |
| A:P ratio | 5.36 | 5.59 | 0.07 | 0.117 |

**Supplementary Table 2.** Rumen fermentation parameters and body weight in pregnant heifers grazing tropical forage (dry season), 6 weeks on 3-NOP (H+) and placebo (H-) treatment.

|  | H- | H+ | SEM | P-value |
| --- | --- | --- | --- | --- |
| BW kg | 564 | 560 | 10.3 | 0.845 |
| Rumen pH | 7.32 | 7.09 | 0.06 | 0.045 |
| Formate mM | 0.04 | 1.48 | 0.31 | 0.023 |
| Lactate mM | 0.020 | 0.013 | 0.004 | 0.435 |
| Succinate mM | 0.011 | 0.015 | 0.004 | 0.531 |
| Fumarate mM | 0.0011 | 0.0002 | 0.00 | 0.003 |
| Ammonia-N mg /100 mL | 3.61 | 4.60 | 0.20 | 0.015 |
| Total VFA mM | 68.0 | 70.3 | 2.37 | 0.635 |
| acetate % | 74.5 | 68.0 | 0.41 | 0.001 |
| propionate % | 14.4 | 17.7 | 0.19 | 0.001 |
| iso-butyrate % | 0.56 | 0.49 | 0.01 | 0.004 |
| n-butyrate % | 8.75 | 11.2 | 0.21 | 0.001 |
| iso-valerate % | 0.80 | 1.13 | 0.04 | 0.001 |
| n-valerate % | 0.65 | 0.99 | 0.03 | 0.001 |
| n-caproate % | 0.33 | 0.54 | 0.03 | 0.003 |
| A:P ratio | 5.19 | 3.90 | 0.07 | 0.001 |

**Supplementary Table 3.** Rumen fermentation parameters and body weight in pregnant heifers grazing tropical forage (dry season), 18 weeks on 3-NOP (H+) and placebo (H-) treatment.

|  | H- | H+ | SEM | P-value |
| --- | --- | --- | --- | --- |
| BW kg | 474 | 479 | 8.65 | 0.757 |
| Rumen pH | 7.27 | 7.36 | 0.06 | 0.477 |
| Formate mM | 0.01 | 0.44 | 0.16 | 0.181 |
| Lactate mM | 0.037 | 0.036 | 0.01 | 0.933 |
| Succinate mM | 0.079 | 0.099 | 0.02 | 0.579 |
| Fumarate mM | 0.002 | 0.003 | 0.001 | 0.794 |
| Ammonia-N mg /100 mL | 2.05 | 3.41 | 0.22 | 0.003 |
| Total VFA mM | 63.7 | 63.3 | 2.76 | 0.936 |
| acetate % | 74.0 | 71.0 | 0.28 | 0.001 |
| propionate % | 15.4 | 16.8 | 0.15 | 0.001 |
| iso-butyrate % | 0.46 | 0.50 | 0.01 | 0.012 |
| n-butyrate % | 8.76 | 9.75 | 0.17 | 0.006 |
| iso-valerate % | 0.52 | 0.73 | 0.02 | 0.001 |
| n-valerate % | 0.56 | 0.71 | 0.02 | 0.001 |
| n-caproate % | 0.27 | 0.41 | 0.02 | 0.001 |
| A:P ratio | 4.82 | 4.24 | 0.05 | 0.001 |

**Supplementary Table 4.** Rumen fermentation parameters and body weight in pregnant heifers grazing tropical forage (late dry season), 23 weeks on 3-NOP (H+) and placebo (H-) treatment.

|  | H- | H+ | SEM | P-value |
| --- | --- | --- | --- | --- |
| BW kg | 421 | 414 | 7.95 | 0.670 |
| Rumen pH | 6.91 | 6.89 | 0.06 | 0.854 |
| Formate mM | 0.02 | 2.93 | 0.52 | 0.007 |
| Lactate mM | 0.05 | 0.07 | 0.01 | 0.401 |
| Succinate mM | 0.06 | 0.15 | 0.02 | 0.007 |
| Fumarate mM | 0.000 | 0.767 | 0.25 | 0.139 |
| Ammonia-N mg /100 mL | 1.34 | 0.87 | 0.13 | 0.083 |
| Total VFA mM | 71.2 | 69.8 | 2.58 | 0.784 |
| acetate % | 74.1 | 66.3 | 0.40 | 0.001 |
| propionate % | 15.3 | 18.6 | 0.25 | 0.001 |
| iso-butyrate % | 0.50 | 0.53 | 0.01 | 0.029 |
| n-butyrate % | 8.51 | 11.9 | 0.17 | 0.001 |
| iso-valerate % | 0.63 | 1.29 | 0.05 | 0.001 |
| n-valerate % | 0.60 | 0.85 | 0.02 | 0.001 |
| n-caproate % | 0.30 | 0.61 | 0.02 | 0.001 |
| A:P ratio | 4.85 | 3.63 | 0.07 | 0.001 |

**Supplementary Table 5.** Rumen fermentation parameters and body weight in pregnant heifers grazing tropical forage (early wet season), 30 weeks on 3-NOP (H+) and placebo (H-) treatment.

|  | H- | H+ | SEM | P-value |
| --- | --- | --- | --- | --- |
| BW kg | 436 | 425 | 8.29 | 0.511 |
| Rumen pH | 6.92 | 6.81 | 0.04 | 0.200 |
| Formate mM | 0.00 | 1.14 | 0.19 | 0.005 |
| Lactate mM | 0.03 | 0.04 | 0.01 | 0.695 |
| Succinate mM | 0.36 | 0.51 | 0.04 | 0.040 |
| Fumarate mM | 0.001 | 0.003 | 0.00 | 0.016 |
| Ammonia-N mg /100 mL | 10.2 | 9.29 | 0.46 | 0.325 |
| Total VFA mM | 84.1 | 70.8 | 3.63 | 0.075 |
| acetate % | 67.7 | 63.4 | 0.43 | 0.001 |
| propionate % | 14.8 | 17.1 | 0.25 | 0.001 |
| iso-butyrate % | 1.25 | 1.00 | 0.06 | 0.037 |
| n-butyrate % | 13.5 | 15.9 | 0.23 | 0.001 |
| iso-valerate % | 1.61 | 1.41 | 0.05 | 0.055 |
| n-valerate % | 0.84 | 0.85 | 0.03 | 0.840 |
| n-caproate % | 0.24 | 0.31 | 0.02 | 0.161 |
| A:P ratio | 4.62 | 3.77 | 0.08 | 0.001 |

**Supplementary table 6.** Calf treatment (3-NOP (C+) or placebo (C-)) effects on rumen fermentation parameters and body weight in 5 weeks old calves.

|  | C- | C+ | SEM | P-value |
| --- | --- | --- | --- | --- |
| BW (Kg) | 65.0 | 68.5 | 1.60 | 0.291 |
| Total VFA mM | 48.0 | 49.0 | 2.03 | 0.797 |
| acetate % | 69.7 | 64.4 | 1.23 | 0.035 |
| propionate % | 20.9 | 25.0 | 0.82 | 0.017 |
| iso-butyrate % | 0.47 | 0.24 | 0.05 | 0.030 |
| n-butyrate % | 6.87 | 8.24 | 0.58 | 0.239 |
| iso-valerate % | 0.82 | 0.91 | 0.13 | 0.722 |
| n-valerate % | 0.92 | 0.97 | 0.10 | 0.829 |
| n-caproate % | 0.27 | 0.30 | 0.02 | 0.552 |
| A:P ratio | 3.59 | 2.79 | 0.19 | 0.038 |

**Supplementary table 7.** Calf treatment (3-NOP (C+) or placebo (C-)) effects on rumen fermentation parameters and body weight in 10 weeks old calves.

|  | C- | C+ | SEM | P-value |
| --- | --- | --- | --- | --- |
| BW (Kg) | 106 | 108 | 2.01 | 0.531 |
| Formate mM | 0.04 | 0.49 | 0.15 | 0.147 |
| Lactate mM | 0.058 | 0.075 | 0.01 | 0.441 |
| Succinate mM | 0.122 | 0.155 | 0.02 | 0.379 |
| Fumarate mM | 0.0004 | 0.0002 | 0.00 | 0.252 |
| Ammonia-N mg /100 mL | 1.30 | 1.07 | 0.16 | 0.454 |
| Total VFA mM | 58.4 | 47.8 | 2.44 | 0.036 |
| acetate % | 68.0 | 63.4 | 0.80 | 0.006 |
| propionate % | 18.4 | 22.2 | 0.62 | 0.004 |
| iso-butyrate % | 0.60 | 0.62 | 0.02 | 0.609 |
| n-butyrate % | 10.5 | 10.8 | 0.31 | 0.629 |
| iso-valerate % | 0.80 | 1.71 | 0.11 | 0.000 |
| n-valerate % | 0.67 | 0.88 | 0.03 | 0.000 |
| n-caproate % | 0.30 | 0.43 | 0.04 | 0.109 |
| A:P ratio | 3.78 | 3.04 | 0.11 | 0.002 |

**Supplementary table 8.** Calf treatment (3-NOP (C+) or placebo (C-)) effects on rumen fermentation parameters and body weight in 15 weeks old calves.

|  | C- | C+ | SEM | P-value |
| --- | --- | --- | --- | --- |
| BW kg | 148 | 151 | 2.87 | 0.668 |
| Formate mM | 0.19 | 2.18 | 0.48 | 0.044 |
| Lactate mM | 0.19 | 0.09 | 0.06 | 0.427 |
| Succinate mM | 0.35 | 0.63 | 0.07 | 0.050 |
| Fumarate Mm | 0.003 | 0.001 | 0.00 | 0.034 |
| Ammonia-N mg /100 mL | 5.24 | 3.73 | 0.76 | 0.326 |
| Total VFA mM | 63.0 | 43.6 | 3.15 | 0.004 |
| acetate % | 69.9 | 63.0 | 0.56 | 0.001 |
| propionate % | 15.8 | 19.0 | 0.51 | 0.003 |
| iso-butyrate % | 0.85 | 0.82 | 0.06 | 0.811 |
| n-butyrate % | 11.2 | 13.9 | 0.29 | 0.001 |
| iso-valerate % | 1.14 | 1.71 | 0.09 | 0.003 |
| n-valerate % | 0.75 | 0.97 | 0.03 | 0.001 |
| n-caproate % | 0.35 | 0.60 | 0.03 | 0.001 |
| A:P ratio | 4.61 | 3.42 | 0.12 | 0.001 |

**Supplementary table 9.** Calf treatment (3-NOP (C+) or placebo (C-)) effects on rumen fermentation parameters and body weight in 22 weeks old calves.

|  | C- | C+ | SEM | P-value |
| --- | --- | --- | --- | --- |
| BW (Kg) | 200 | 205 | 3.56 | 0.537 |
| Formate mM | 0.14 | 0.84 | 0.14 | 0.018 |
| Lactate mM | 0.015 | 0.020 | 0.01 | 0.648 |
| Succinate mM | 0.21 | 0.31 | 0.06 | 0.398 |
| Fumarate Mm | 0.0006 | 0.0008 | 0.00 | 0.436 |
| Ammonia-N mg /100 mL | 6.40 | 4.59 | 0.41 | 0.031 |
| Total VFA mM | 47.3 | 41.2 | 3.03 | 0.322 |
| acetate % | 69.0 | 64.6 | 0.49 | 0.001 |
| propionate % | 13.7 | 16.1 | 0.26 | 0.001 |
| iso-butyrate % | 1.31 | 1.13 | 0.05 | 0.056 |
| n-butyrate % | 12.9 | 14.5 | 0.30 | 0.010 |
| iso-valerate % | 1.62 | 1.96 | 0.05 | 0.003 |
| n-valerate % | 0.90 | 1.05 | 0.02 | 0.001 |
| n-caproate % | 0.44 | 0.66 | 0.04 | 0.004 |
| A:P ratio | 5.07 | 4.12 | 0.11 | 0.001 |

**Supplementary table 10.** Rumen fermentation parameters and body weight in 35 weeks old weaners, 12 weeks post-treatment with 3-NOP (W+) or placebo (W-). Animals from 1^st^ calving group.

|  | W- | W+ | SEM | P-value |
| --- | --- | --- | --- | --- |
| BW kg | 253 | 260 | 5.73 | 0.567 |
| Rumen pH | 7.13 | 7.27 | 0.05 | 0.179 |
| Formate mM | 0.22 | 0.20 | 0.002 | 0.782 |
| Lactate mM | 0.009 | 0.013 | 0.003 | 0.416 |
| Succinate mM | 0.037 | 0.029 | 0.005 | 0.478 |
| Fumarate Mm | 0.0018 | 0.0014 | 0.000 | 0.581 |
| Ammonia-N mg /100 mL | 6.55 | 6.56 | 0.35 | 0.981 |
| Total VFA mM | 49.8 | 44.2 | 3.31 | 0.407 |
| acetate % | 76.0 | 76.3 | 0.35 | 0.662 |
| propionate % | 11.9 | 11.7 | 0.13 | 0.402 |
| iso-butyrate % | 0.94 | 0.99 | 0.05 | 0.651 |
| n-butyrate % | 9.31 | 9.11 | 0.19 | 0.609 |
| iso-valerate % | 1.08 | 1.12 | 0.04 | 0.593 |
| n-valerate % | 0.72 | 0.74 | 0.04 | 0.777 |
| A:P ratio | 6.41 | 6.56 | 0.10 | 0.446 |

**Supplementary table 11.** Rumen fermentation parameters and body weight in 35 weeks old weaners, 12 weeks post-treatment with 3-NOP (W+) or placebo (W-). Animals from 2^nd^ calving group.

|  | W- | W+ | SEM | P-value |
| --- | --- | --- | --- | --- |
| BW kg | 253 | 257 | 7.58 | 0.806 |
| Rumen pH | 6.99 | 7.14 | 0.05 | 0.149 |
| Formate mM | 0.001 | 0.019 | 0.003 | 0.010 |
| Lactate mM | 0.001 | 0.010 | 0.002 | 0.026 |
| Succinate mM | 0.125 | 0.180 | 0.004 | 0.521 |
| Fumarate Mm | 0.0007 | 0.0019 | 0.000 | 0.020 |
| Ammonia-N mg /100 mL | 7.31 | 7.03 | 0.50 | 0.786 |
| Total VFA mM | 55.0 | 46.5 | 1.97 | 0.046 |
| acetate % | 67.6 | 71.4 | 0.66 | 0.011 |
| propionate % | 17.3 | 14.6 | 0.31 | 0.001 |
| iso-butyrate % | 1.13 | 1.16 | 0.06 | 0.841 |
| n-butyrate % | 11.7 | 10.8 | 0.32 | 0.156 |
| iso-valerate % | 1.16 | 1.13 | 0.06 | 0.786 |
| n-valerate % | 0.89 | 0.71 | 0.04 | 0.031 |
| n-caproate % | 0.22 | 0.17 | 0.01 | 0.076 |
| A:P ratio | 3.93 | 4.90 | 0.13 | 0.002 |

**Supplementary table 12.** Rumen fermentation parameters and body weight in 55 weeks old weaners, 28 weeks post-treatment with 3-NOP (W+) or placebo (W-). Animals from 1^st^ calving group.

|  | W- | W+ | SEM | P-value |
| --- | --- | --- | --- | --- |
| BW kg | 322 | 325 | 6.80 | 0.842 |
| Rumen pH | 7.63 | 7.65 | 0.05 | 0.822 |
| Formate mM | 0.045 | 0.060 | 0.010 | 0.441 |
| Lactate mM | 0.009 | 0.014 | 0.004 | 0.476 |
| Succinate mM | 0.024 | 0.007 | 0.008 | 0.271 |
| Fumarate Mm | 0.0009 | 0.0017 | 0.000 | 0.329 |
| Ammonia-N mg /100 mL | 4.86 | 4.20 | 0.32 | 0.305 |
| Total VFA mM | 51.7 | 42.1 | 3.24 | 0.150 |
| acetate % | 74.4 | 74.7 | 0.14 | 0.262 |
| propionate % | 14.6 | 14.6 | 0.12 | 0.901 |
| iso-butyrate % | 0.66 | 0.59 | 0.05 | 0.495 |
| n-butyrate % | 8.89 | 8.58 | 0.10 | 0.153 |
| iso-valerate % | 0.71 | 0.69 | 0.03 | 0.578 |
| n-valerate % | 0.59 | 0.58 | 0.02 | 0.771 |
| n-caproate % | 0.14 | 0.20 | 0.03 | 0.297 |
| A:P ratio | 5.10 | 5.11 | 0.05 | 0.927 |

**Supplementary table 13.** Rumen fermentation parameters and body weight in 55 weeks old weaners, 28 weeks post-treatment with 3-NOP (W+) or placebo (W-). Animals from 2^nd^ calving group.

|  | W- | W+ | SEM | P-value |
| --- | --- | --- | --- | --- |
| BW kg | 277 | 273 | 8.69 | 0.834 |
| Rumen pH | 7.11 | 7.00 | 0.04 | 0.192 |
| Formate mM | 0.07 | 0.07 | 0.008 | 0.999 |
| Lactate mM | 0.02 | 0.03 | 0.004 | 0.256 |
| Succinate mM | 0.02 | 0.01 | 0.003 | 0.583 |
| Fumarate Mm | 0.014 | 0.013 | 0.000 | 0.863 |
| Ammonia-N mg /100 mL | 2.62 | 2.62 | 0.16 | 0.989 |
| Total VFA mM | 43.7 | 46.4 | 3.31 | 0.680 |
| acetate % | 74.8 | 74.6 | 0.19 | 0.688 |
| propionate % | 14.7 | 15.0 | 0.12 | 0.304 |
| iso-butyrate % | 0.39 | 0.39 | 0.01 | 0.873 |
| n-butyrate % | 9.14 | 8.96 | 0.13 | 0.514 |
| iso-valerate % | 0.44 | 0.49 | 0.02 | 0.245 |
| n-valerate % | 0.42 | 0.42 | 0.01 | 0.806 |
| n-caproate % | 0.11 | 0.11 | 0.01 | 0.986 |
| A:P ratio | 5.09 | 4.99 | 0.05 | 0.341 |

**Supplementary table 14.** Dam effect (H- or H+) on rumen fermentation parameters and body weight in untreated calves (5 weeks old).

|  | H- C- | H+ C- | SEM | P-value |
| --- | --- | --- | --- | --- |
| BW (Kg) | 67.3 | 63.6 | 2.259 | 0.425 |
| Total VFA mM | 47.7 | 45.0 | 3.452 | 0.702 |
| acetate % | 69.0 | 70.9 | 1.545 | 0.548 |
| propionate % | 21.7 | 20.3 | 0.965 | 0.463 |
| iso-butyrate % | 0.34 | 0.55 | 0.080 | 0.210 |
| n-butyrate % | 7.08 | 6.27 | 0.628 | 0.527 |
| iso-valerate % | 0.72 | 0.87 | 0.078 | 0.372 |
| n-valerate % | 0.84 | 0.95 | 0.118 | 0.637 |
| n-caproate % | 0.34 | 0.24 | 0.037 | 0.193 |
| A:P ratio | 3.28 | 3.58 | 0.263 | 0.276 |

**Supplementary table 15.** Dam effect (H- or H+) on rumen fermentation parameters and body weight in untreated calves (10 weeks old).

|  | H- C- | H+ C- | SEM | P-value |
| --- | --- | --- | --- | --- |
| BW (Kg) | 109 | 102 | 2.989 | 0.232 |
| Formate mM | 0.07 | 0.02 | 0.017 | 0.230 |
| Lactate mM | 0.08 | 0.04 | 0.016 | 0.195 |
| Succinate mM | 0.10 | 0.14 | 0.019 | 0.252 |
| Fumarate mM | 0.0002 | 0.0005 | 0.000 | 0.114 |
| Ammonia-N mg /100 mL | 1.40 | 1.21 | 0.246 | 0.701 |
| Total VFA mM | 56.4 | 60.4 | 3.233 | 0.542 |
| acetate % | 67.9 | 68.1 | 0.910 | 0.921 |
| propionate % | 19.2 | 17.6 | 0.589 | 0.188 |
| iso-butyrate % | 0.58 | 0.62 | 0.026 | 0.480 |
| n-butyrate % | 10.7 | 10.2 | 0.327 | 0.527 |
| iso-valerate % | 0.71 | 0.88 | 0.075 | 0.246 |
| n-valerate % | 0.67 | 0.66 | 0.036 | 0.947 |
| n-caproate % | 0.29 | 0.31 | 0.030 | 0.805 |
| A:P ratio | 3.63 | 3.93 | 0.131 | 0.264 |

**Supplementary table 16.** Dam effect (H- or H+) on rumen fermentation parameters and body weight in untreated calves (15 weeks old).

|  | H- C- | H+ C- | SEM | P-value |
| --- | --- | --- | --- | --- |
| BW kg | 150 | 151 | 4.266 | 0.873 |
| Formate mM | 0.19 | 0.10 | 0.033 | 0.165 |
| Lactate mM | 0.047 | 0.48 | 0.148 | 0.164 |
| Succinate mM | 0.35 | 0.47 | 0.102 | 0.562 |
| Fumarate Mm | 0.0007 | 0.0051 | 0.001 | 0.015 |
| Ammonia-N mg /100 mL | 4.09 | 9.61 | 1.664 | 0.116 |
| Total VFA mM | 60.6 | 67.6 | 4.573 | 0.454 |
| acetate % | 68.8 | 70.0 | 1.054 | 0.581 |
| propionate % | 17.0 | 14.4 | 0.993 | 0.212 |
| iso-butyrate % | 0.84 | 1.05 | 0.108 | 0.347 |
| n-butyrate % | 11.2 | 11.8 | 0.468 | 0.528 |
| iso-valerate % | 1.11 | 1.47 | 0.175 | 0.319 |
| n-valerate % | 0.75 | 0.86 | 0.062 | 0.412 |
| n-caproate % | 0.32 | 0.44 | 0.037 | 0.142 |
| A:P ratio | 4.32 | 4.92 | 0.215 | 0.184 |

**Supplementary table 17.** Dam effect (H- or H+) on rumen fermentation parameters and body weight in untreated calves (22 weeks old).

|  | H- C- | H+ C- | SEM | P-value |
| --- | --- | --- | --- | --- |
| BW (Kg) | 202 | 198 | 4.903 | 0.584 |
| Formate mM | 0.24 | 0.04 | 0.094 | 0.303 |
| Lactate mM | 0.017 | 0.014 | 0.007 | 0.805 |
| Succinate mM | 0.25 | 0.17 | 0.047 | 0.403 |
| Fumarate Mm | 0.0004 | 0.0007 | 0.000 | 0.440 |
| Ammonia-N mg /100 mL | 5.91 | 6.90 | 0.760 | 0.522 |
| Total VFA mM | 49.8 | 44.7 | 4.627 | 0.588 |
| acetate % | 69.8 | 68.3 | 0.398 | 0.084 |
| propionate % | 14.0 | 13.5 | 0.265 | 0.316 |
| iso-butyrate % | 1.25 | 1.38 | 0.068 | 0.370 |
| n-butyrate % | 12.1 | 13.7 | 0.305 | 0.015 |
| iso-valerate % | 1.57 | 1.68 | 0.077 | 0.502 |
| n-valerate % | 0.83 | 0.97 | 0.022 | 0.006 |
| n-caproate % | 0.44 | 0.44 | 0.042 | 0.998 |
| A:P ratio | 5.01 | 5.13 | 0.124 | 0.626 |

**Supplementary table 18.** Dam effect (H- or H+) on rumen fermentation parameters and body weight in untreated weaners (35 weeks old), 3 months post-weaning.

|  | H- W- | H+ W- | SEM | P-value |
| --- | --- | --- | --- | --- |
| BW kg | 261 | 244 | 7.864 | 0.301 |
| Methane g/day | 205 | 210 | 4.774 | 0.619 |
| Formate mM | 0.011 | 0.018 | 0.003 | 0.263 |
| Lactate mM | 0.007 | 0.005 | 0.001 | 0.352 |
| Succinate mM | 0.027 | 0.030 | 0.006 | 0.755 |
| Fumarate Mm | 0.001 | 0.002 | 0.000 | 0.120 |
| Ammonia-N mg /100 mL | 6.90 | 6.69 | 0.384 | 0.790 |
| Total VFA mM | 52.5 | 50.6 | 3.192 | 0.766 |
| acetate % | 72.9 | 73.6 | 1.038 | 0.738 |
| propionate % | 14.0 | 13.7 | 0.621 | 0.642 |
| iso-butyrate % | 1.00 | 1.01 | 0.054 | 0.883 |
| n-butyrate % | 10.2 | 10.0 | 0.333 | 0.844 |
| iso-valerate % | 1.10 | 1.11 | 0.059 | 0.918 |
| n-valerate % | 0.77 | 0.78 | 0.035 | 0.953 |
| A:P ratio | 5.50 | 5.67 | 0.287 | 0.766 |

**Supplementary table 19.** Dam effect (H- or H+) on rumen fermentation parameters and body weight in untreated weaners (55 weeks old), 8 months post-weaning.

|  | H- W- | H+ W- | SEM | P-value |
| --- | --- | --- | --- | --- |
| BW kg | 315 | 298 | 3.354 | 0.427 |
| Methane g/day | 156 | 163 | 4.574 | 0.464 |
| Formate mM | 0.051 | 0.053 | 0.011 | 0.933 |
| Lactate mM | 0.012 | 0.013 | 0.004 | 0.846 |
| Succinate mM | 0.014 | 0.030 | 0.009 | 0.411 |
| Fumarate Mm | 0.001 | 0.001 | 0.000 | 0.468 |
| Ammonia-N mg /100 mL | 4.33 | 3.88 | 0.360 | 0.534 |
| Total VFA mM | 51.6 | 46.2 | 3.354 | 0.427 |
| acetate % | 74.4 | 74.7 | 0.135 | 0.166 |
| propionate % | 14.6 | 14.7 | 0.131 | 0.949 |
| iso-butyrate % | 0.57 | 0.57 | 0.056 | 0.998 |
| n-butyrate % | 9.08 | 8.85 | 0.109 | 0.288 |
| iso-valerate % | 0.64 | 0.61 | 0.038 | 0.672 |
| n-valerate % | 0.58 | 0.48 | 0.025 | 0.083 |
| n-caproate % | 0.15 | 0.11 | 0.019 | 0.264 |
| A:P ratio | 5.09 | 5.11 | 0.052 | 0.863 |


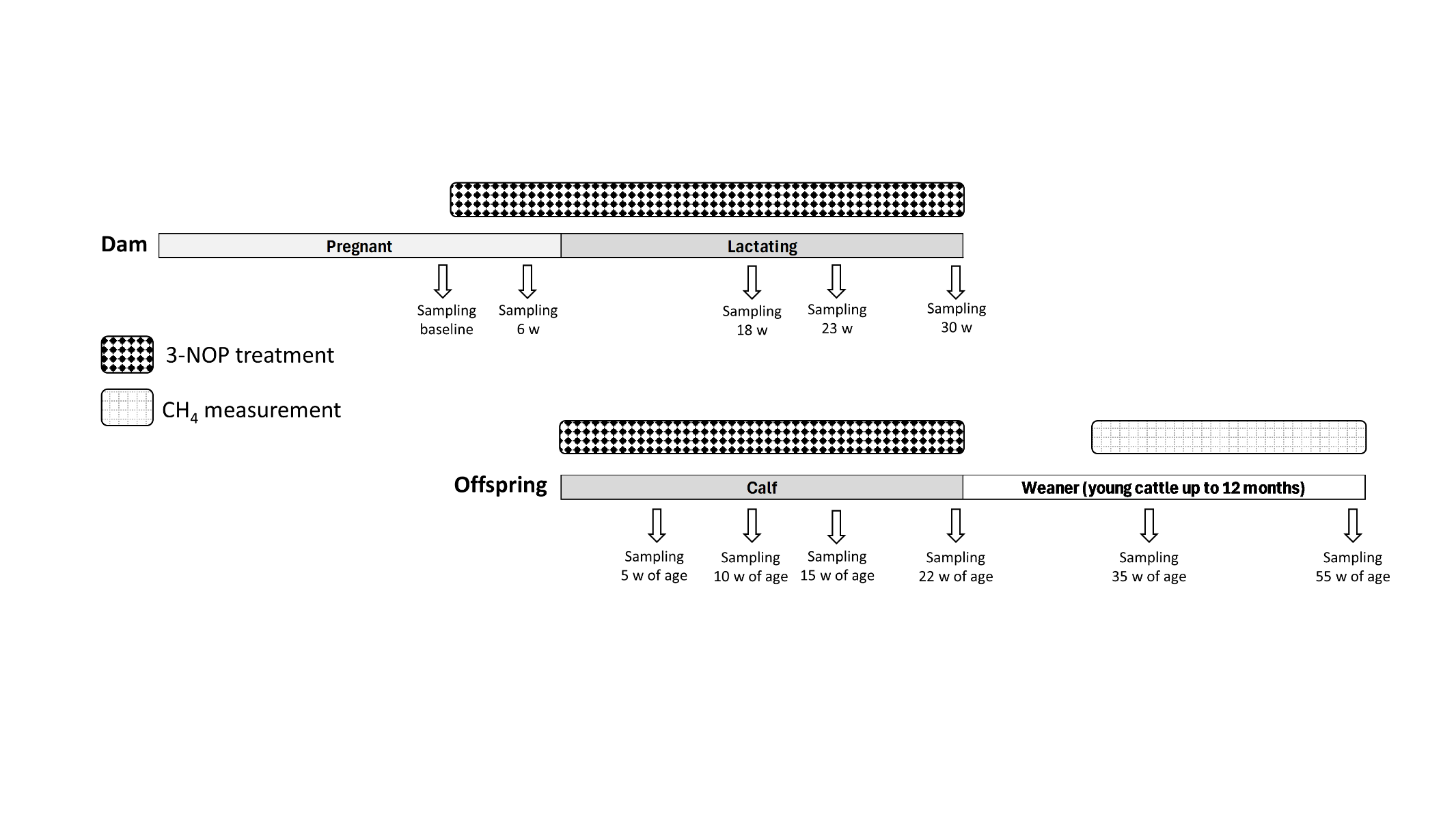


Supplementary Figure 1. Trial timeline and sampling events.

C)

B)

A)

D)

**Supplementary Figure 2.** Quantitative PCR analysis of total methanogens (McrA gene), *Methanobrevibacter* and *Methanomassiliicoccaceae* population changes in response to 3-NOP supplementation in heifers (H+) at 6 (A), 18 (B), 23 (C) and 30 (D) weeks on treatment. The y-axis denotes fold change from control group (H-); *** denote significant differences between treatment and control ( P < 0.001); ** denote significant differences between treatment and control ( P < 0.01); *t* denote a trend between treatment and control ( P < 0.1).

D)

C)

B)

A)

**Supplementary Figure 3.** Quantitative PCR analysis of total methanogens (McrA gene), *Methanobrevibacter* and *Methanomassiliicoccaceae* population changes in response to 3-NOP supplementation in calves (C+) at 5 (A), 10 (B), 15 (C) and 22 (D) weeks old. The y-axis denotes fold change from untreated calves (C-); *** denote significant differences between treatment and control ( P < 0.001); ** denote significant differences between treatment and control ( P < 0.01); * denote a trend between treatment and control ( P < 0.05).
